# Genome-wide features of neuroendocrine regulation in *Drosophila* by the basic helix-loop-helix transcription factor DIMMED

**DOI:** 10.1093/nar/gku1377

**Published:** 2015-01-29

**Authors:** Tarik Hadžić, Dongkook Park, Katharine C. Abruzzi, Lin Yang, Jennifer S. Trigg, Remo Rohs, Michael Rosbash, Paul H. Taghert

**Affiliations:** 1 Department of Anatomy and Neurobiology, Washington University School of Medicine, 660 South Euclid Avenue, St. Louis, MO 63110, USA; 2 Howard Hughes Medical Institute, National Center for Behavioral Genomics, Department of Biology, Brandeis University, Waltham, MA 02454, USA; 3 Molecular and Computational Biology Program, Department of Biological Sciences, University of Southern California, Los Angeles, CA 90089, USA

## Abstract

Neuroendocrine (NE) cells use large dense core vesicles (LDCVs) to traffic, process, store and secrete neuropeptide hormones through the regulated secretory pathway. The dimmed (DIMM) basic helix-loop-helix transcription factor of *Drosophila* controls the level of regulated secretory activity in NE cells. To pursue its mechanisms, we have performed two independent genome-wide analyses of DIMM's activities: (i) *in vivo* chromatin immunoprecipitation (ChIP) to define genomic sites of DIMM occupancy and (ii) deep sequencing of purified DIMM neurons to characterize their transcriptional profile. By this combined approach, we showed that DIMM binds to conserved E-boxes in enhancers of 212 genes whose expression is enriched in DIMM-expressing NE cells. DIMM binds preferentially to certain E-boxes within first introns of specific gene isoforms. Statistical machine learning revealed that flanking regions of putative DIMM binding sites contribute to its DNA binding specificity. DIMM's transcriptional repertoire features at least 20 LDCV constituents. In addition, DIMM notably targets the pro-secretory transcription factor, *creb-A*, but significantly, DIMM does not target any neuropeptide genes. DIMM therefore prescribes the scale of secretory activity in NE neurons, by a systematic control of both proximal and distal points in the regulated secretory pathway.

## INTRODUCTION

In the mammalian hypothalamus, a small cohort of neuroendocrine (NE) neurons produces the peptide hormones vasopressin (VP) or oxytocin (OX) for release into the blood via the hypophysial portal system. Peptides like VP and OX must travel long distances, in sufficient quantities and at the appropriate times to deliver organism-critical physiological information. Thus to perform their cellular functions, NE neurons like the VP- and OX-expressing neurons must coordinate and respond to a complex set of physiological, cellular and biochemical requirements (1). They must produce large amounts of secretory products and balance that demand with a comparably high secretory capacity. Furthermore, they must place those secretory products in a readily releasable storage compartment and then recognize and respond to specific triggering stimuli, by releasing a large fraction of the stored peptide in a short period of time. Finally, that cycle of synthesis, storage and accurately timed release must begin anew and proceed in phase with the previous cycle of release.

These complex, high volume and highly choreographed features of regulated protein secretion are not generalized cellular properties. Instead, a series of molecular and genetic studies *in vitro* and *in vivo* suggest that such cellular properties result from sophisticated and cell-intrinsic genetic programs (2,3,4). Several transcription factors play instrumental roles in these programs, including: (i) XBP1 that promotes expansion of the rough endoplasmic reticulum (ER), regulates the unfolded protein response and may be required for differentiation of dedicated secretory cells such as antibody-secreting plasma cells and intestinal Paneth cells (5); (ii) REST that in PC12 cells governs a suite of genes that coordinately support neurosecretion (6); and (iii) the CREB-A protein that in *Drosophila* promotes a pro-secretory program in constitutively secreting tissues like the salivary glands (7).

In *Drosophila* NE neurons, the basic helix-loop-helix (bHLH) TF DIMM is an important component of this genetic regulatory program. By virtue of its normal expression, and its loss-of-function phenotypes, we have proposed that DIMM is required to support the dedicated, high secretory capacity of NE neurons that produce many diverse NE peptides (8,9,10,11). Its mammalian ortholog, Mist1, is a comparable regulator of high secretory capacity in serous (protein-secreting) exocrine cells (12,13). DIMM and Mist1 are prototypical scaling factors (14): they specify the quantitative features of cellular phenotype, and not its qualitative aspects (i.e. they are not cell fate determinants).

Neuropeptides and peptide hormones are processed and stored within specialized large dense-core vesicles (LDCVs) (15,16). DIMM is a pro-LDCV regulatory factor: when it is mis-expressed in conventional (i.e. non-peptidergic) neurons, it efficiently converts them to an NE function (17). That transformation includes the dramatic downregulation of synaptic active zones and of small synaptic vesicles, and the remarkable promotion of functional LDCVs. DIMM-dependent LDCVs are functional because they can store ectopic neuropeptides and because they permit complete post-translational precursor processing. The ability to promote biogenesis of complete sub-cellular organelles parallels the actions of two notable transcriptional regulators. First is the transcriptional coactivator PPARγ coactivator-1 alpha (PGC1α), which promotes commitment of cellular resources to adenosine triphosphate (ATP) production (18). Its mis-expression *in vivo* leads to increased mitochondrial mass in the heart at the expense of other organelles (19), and it activates many key mitochondrial genes (20). Likewise, transcription factor EB (TFEB), a bHLH-leucine zipper TF promotes lysosomal biosynthesis, and directly regulates a network of ∼300 target genes that include numerous structural components of the lysosome and other targets that protect against leakage of proteases from it. Thus transcriptional regulation of entire sub-cellular organelles by Scaling Factors, like PGC1α and TFEB, provides an efficient means by which cellular physiology can be compartmentalized and adapted to changing homeostatic demands.

Our research is directed at understanding the mechanisms by which the DIMM Scaling Factor efficiently coordinates the cellular machinery underlying regulated secretory capacity and the production of specialized LDCVs in NE cells. To pursue that goal, we previously identified a total of ∼10 DIMM direct transcriptional targets using highly focused molecular screens (11,21). That list includes the neuropeptide biosynthetic enzyme *Phm* and the cytochrome b-561 isoform CG1275. The latter is an integral LDCV membrane protein that provides reducing equivalents from the cytosol to the LDCV lumen to replenish the stores of ascorbate (a necessary Peptidylglycine alpha-amidating monooxygenase (PHM) cofactor that is oxidized with each reaction cycle; (22)). To extend that analysis beyond the provisional list of just a few targets, we now report on the use of two independent molecular methods that together provide a genome-wide description of the DIMM transcriptional program. The aims are 2-fold: to create a molecular signature of DIMM binding sites and to provide a broad definition of which genes DIMM targets. In doing so, we sought to produce hypotheses that can describe the scope of DIMM's cellular activities.

## MATERIALS AND METHODS

### Chromatin immunoprecipitation-chip

We performed chromatin immunoprecipitation (ChIP) as previously described (21,23). Briefly, tagged ChIP was carried out with a DIMM::MYC-tag fusion transgene that can rescue diminished neuropeptide levels in DIMM null animals (10). In order to express DIMM::MYC only in adult DIMM-expressing neurons, we used the TARGET system (24). The UAS-*dimm::myc* transgene was combined with a temperature-sensitive *tub*-GAL80*^ts^* element and *c929*-GAL4, which is inserted in a *dimm* enhancer and overlaps almost completely with DIMM protein (8,10). Animals harboring all three elements and control animals lacking UAS-DIMM::MYC developed normally at the restrictive temperature (18°C), with undetectable DIMM::MYC expression (data not shown). Two-to-three-day-old adults were shifted to the permissive temperature (30°C) and specific induction of the DIMM::MYC protein within 72 h was confirmed by western blotting (data not shown). Two sets of animals were used for this experiment: (i) *c929*-GAL4/UAS-*dimm::myc*; *tub*-GAL80*^ts^*/+ (experimental group) and (ii) *c929*-GAL4; *tub*-GAL80*^ts^* maternal strain (negative control). Following crosslink reversal, DNA was processed according to the Affymetrix® Chromatin Immunoprecipitation Assay Protocol (P/N 702238 Rev. 3). For each sample, 10 μl of undiluted ChIP DNA or 1:10-diluted input DNA was amplified by linear polymerase chain reaction (PCR)-based amplification. We used quantitative PCR as described by Menet *et al*. (23). To confirm that, even after amplification, DIMM continued to occupy a known binding site in the target *Phm* (11), 6 μg of each amplified sample was fragmented and biotin-labeled by Terminal Deoxynucleotidyl Transferase, then hybridized to Affymetrix GeneChip® *Drosophila* 2.0 Tiling Arrays and scanned according to Affymetrix protocols at the National Center for Behavioral Genomics (Brandeis University).

### Bioinformatics analysis of ChIP-chip data

To detect statistically significant DIMM binding peaks throughout the genome we used the Model-based Analysis of Tiling-arrays (MAT; (25)). The following settings were used: MAT version 06022009 with BandWidth = 300, MaxGap = 300 and MinProbe = 8. The resulting MAT score, which is calculated from normalized probe intensities, represents a statistical likelihood that a particular genomic region is enriched in the immunoprecipitated relative to the control sample (26). A total of eight tiling arrays were used: two independent biological replicates each, of:

1. *w;c929*-GAL4/UAS-*DIMM::MYC*; *tub*gal80^*ts*^/+ MYC ChIP;
2. *w;c929*-GAL4/UAS-*DIMM::MYC*; tubgal80^ts^/+input DNA;
3. *w;c929*-GAL4; *tub*gal80^*ts*^MYC ChIP;
4. *w;c929*-GAL4; *tub*gal80^*ts*^input DNA.

Thus, an identical number of arrays were used for experimental and control samples, with the same number of biological replicates and DNA amounts hybridized to tiling arrays. Analysis and normalization was carried out as follows: samples from #1 were normalized against input in #2 in MAT. Samples from #3 were normalized against their input in #4 in MAT. In order to provide additional stringency and as a means of independent verification, ChIPed DNA from experimental samples in #1 was normalized directly against ChIPed DNA in controls in #3 in MAT. Next, raw signals from MYC ChIP signal normalized to its input of the control sample (#3–#4) were subtracted from the experimental group MYC ChIP signal normalized to its input (#1–#2). Raw signal derived from normalizing MYC ChIP in the experimental group to MYC ChIP in the control group (#1–#3) was then added to the subtracted signal (#1 – #2) – (#3 – #4). This is represented by the following equation: [(#1 – #2) – (#3 – #4)] + (#1 – #3). We found that signal peaks obtained from (#1 – #2) – (#3 – #4) normalization coincided with peaks from (#1 – #3) normalization in majority of cases. Therefore, we felt that obtaining binding peaks in the same location defined by these two different normalization steps enhanced the data fidelity. For example, the known *in vivo* DIMM target *Phm* demonstrates a distinct signal peak over its first intron that overlaps in the (#1 – #2) and (#1 – #3) normalizations, whereas (#3 – #4) showed no binding (see Supplementary Figure S3). Statistically significant deviations of signal from baseline were calculated for this twice normalized raw signal at *P*-value ≤1 × 10^−4^ with a naïve peak caller algorithm (Cistrome). Visual output from all three MAT analyses performed (DIMM ChIP/Input, NEG ChIP/Input and DIMM ChIP/NEG ChIP) can be directly compared to each other as long as the window minima and maxima are identical.

### Genomic annotation of DIMM ChIP-chip peaks

We annotated DIMM binding sites with Galaxy and Cistrome, using Flybase release 5.50 used for all annotations (27,28). Figure 3B, C, D and F was produced in the CEAS package (29). Figure 4F and G was produced in the SitePro package (29). Figure 6 was produced with the Heatmap tool (Cistrome) and GENE-E (http://www.broadinstitute.org/cancer/software/GENE-E/). All other graphs were produced in OS X R version 3.0.2 GUI 1.62 or in Microsoft Excel 2011. Cistrome SeqPos was used to detect statistically significant DNA binding motifs (28). Found motifs were confirmed with an independent analysis in RSAT (30). For conservation analysis, UCSC Genome Browser dm3 phastCons15way table was used (31). We conducted ontology analysis in GO Elite v1.2.6 (32), and results were visualized in Cytoscape v.3.0.2 (33). For LDCV constituent analysis, we used data from Gauthier *et al*. (34), derived from a proteomic analysis of the corticotropes dense-core secretory granules. Additionally, we used a recently published list of human LDCV constituents derived from proteomic analysis of a human pheochromocytoma sample (35). *Drosophila* orthologs of the human LDCV constituent genes were identified in Flymine (36) and genetic interactions were mined from Flybase (37).

**Figure 1.**
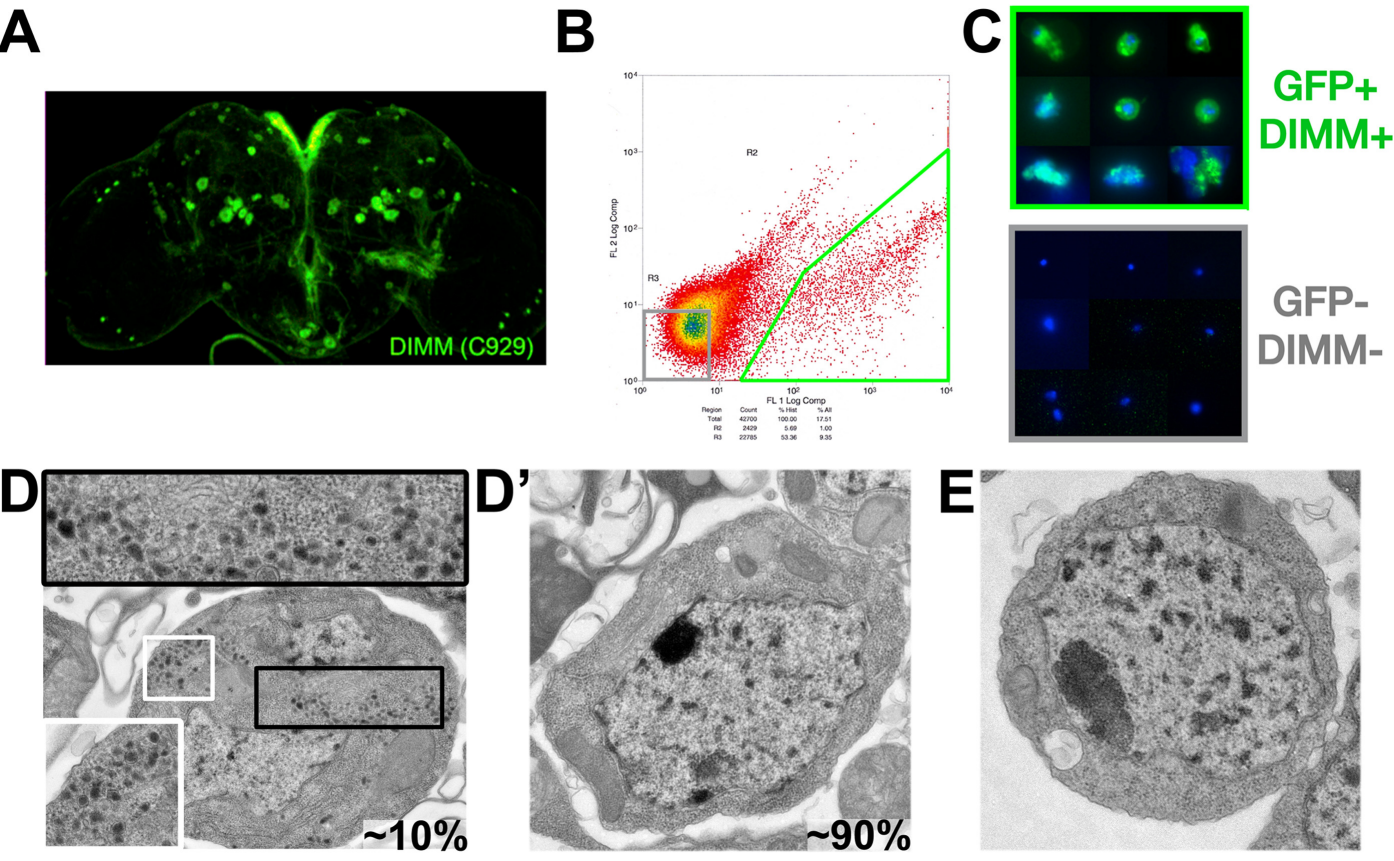
Isolation of *c929* (DIMM^+^) neurons from the adult brain. (**A**) Sample dissected adult brain used for sorting. (**B**) FACS-sorting of DIMM-positive neurons (bottom right) and DIMM-negative randomly sorted neurons (bottom left) from dissected adult fly brains. (**C**) Light microscopy of control gate cells lacking GFP expression (bottom) compared to high fluorescence gate cells (top). (**D, D’**) Electron micrographs depicting cells representative of ∼10 and ∼90%, respectively, of *c929*>GFP^+^ neurons after FACS and EM processing. (**E**) Electron micrograph showing a representative *c929*>GFP^−^ neuron.

**Figure 2.**
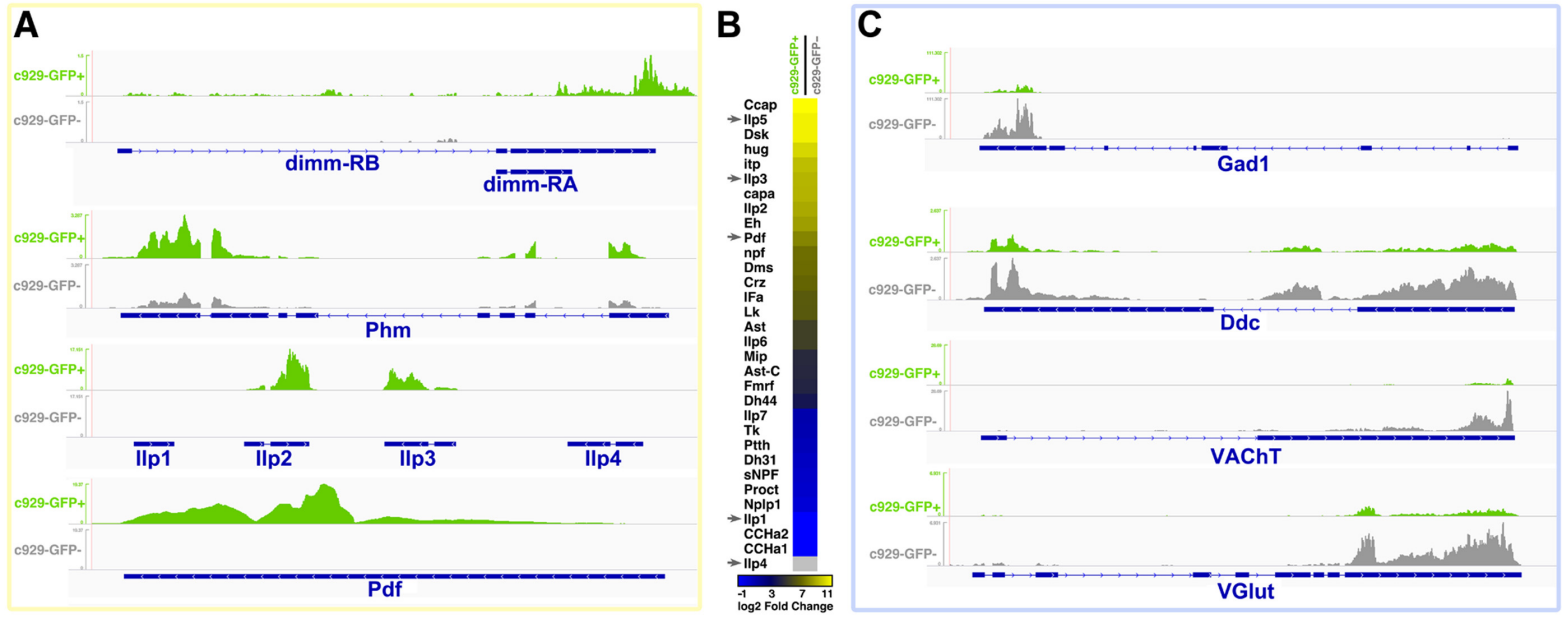
Deep sequencing of DIMM^+^ cell and control cell transcriptomes reveals enrichment of known peptidergic markers and little overlap with markers of major non-peptidergic neuronal subtypes. (**A**) The *c929*>GFP^+^ sample is highly enriched for *dimm* RNA (top). DIMM's direct target *Phm* is ∼3.5-fold enriched in the *c929*-GFP^+^ sample compared to the *c929*-GFP^−^ sample. Likewise the known neurally-expressed bioactive peptides *ilp2, ilp3* and *Pdf* transcripts are all enriched in *c929*-GFP^+^ neurons, while the *ilp1* and *ilp4* transcripts (poorly expressed in the brain) are not detected in either transcriptome. (**B**) Most, but not all, neuropeptide RNAs are strongly enriched in the *c929*-GFP^+^ cells compared to controls. This distribution compares favorably to known neuropeptide distributions among DIMM^+^ neurons (52).(**C**) *c929*-GFP^+^ cells are not enriched for gene markers of GABAergic, dopaminergic, cholinergic and glutamatergic neurons, compared to the *c929*-GFP^−^ cell population.

**Figure 3.**
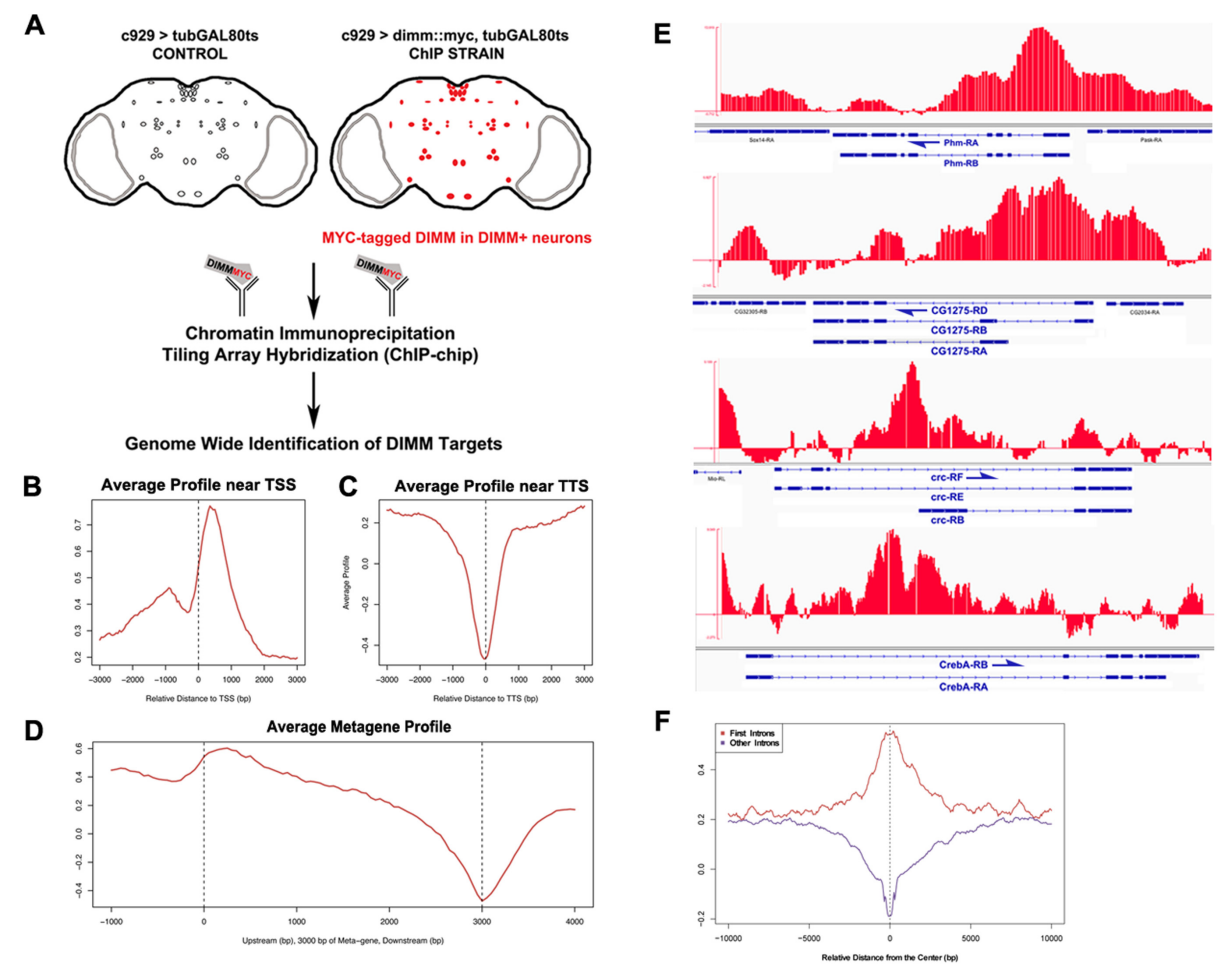
DIMM binds to more than 300 loci. In addition to binding known target genes such as *Phm* and *CG1275*, DIMM binds hundreds of novel targets. (**A**) The schematic depicts genotypes and the experimental setup used for ChIP-chip. (**B**) DIMM binding is enriched near transcription start sites and (**C**) depleted near transcription termination sites. (**D**) A ‘metagene’ profile derived from an average of all genes in the genome shows striking enrichment of DIMM binding in the promoter/5′UTR sites, often peaking in the first 300 bp of a metagene. (**E**) ChIP-chip recapitulates known *in vivo* DIMM binding to the first intron of *Phm*. Novel targets include Creb/ATF family pro-secretory transcription factors *CrebA* and *cryptocephal*, as well as previously uncharacterized genes, such as *CG4577*. (**F**) DIMM binds preferentially to the first introns and transcriptional start sites, whereas DIMM binding over remaining gene introns is depleted.

**Figure 4.**
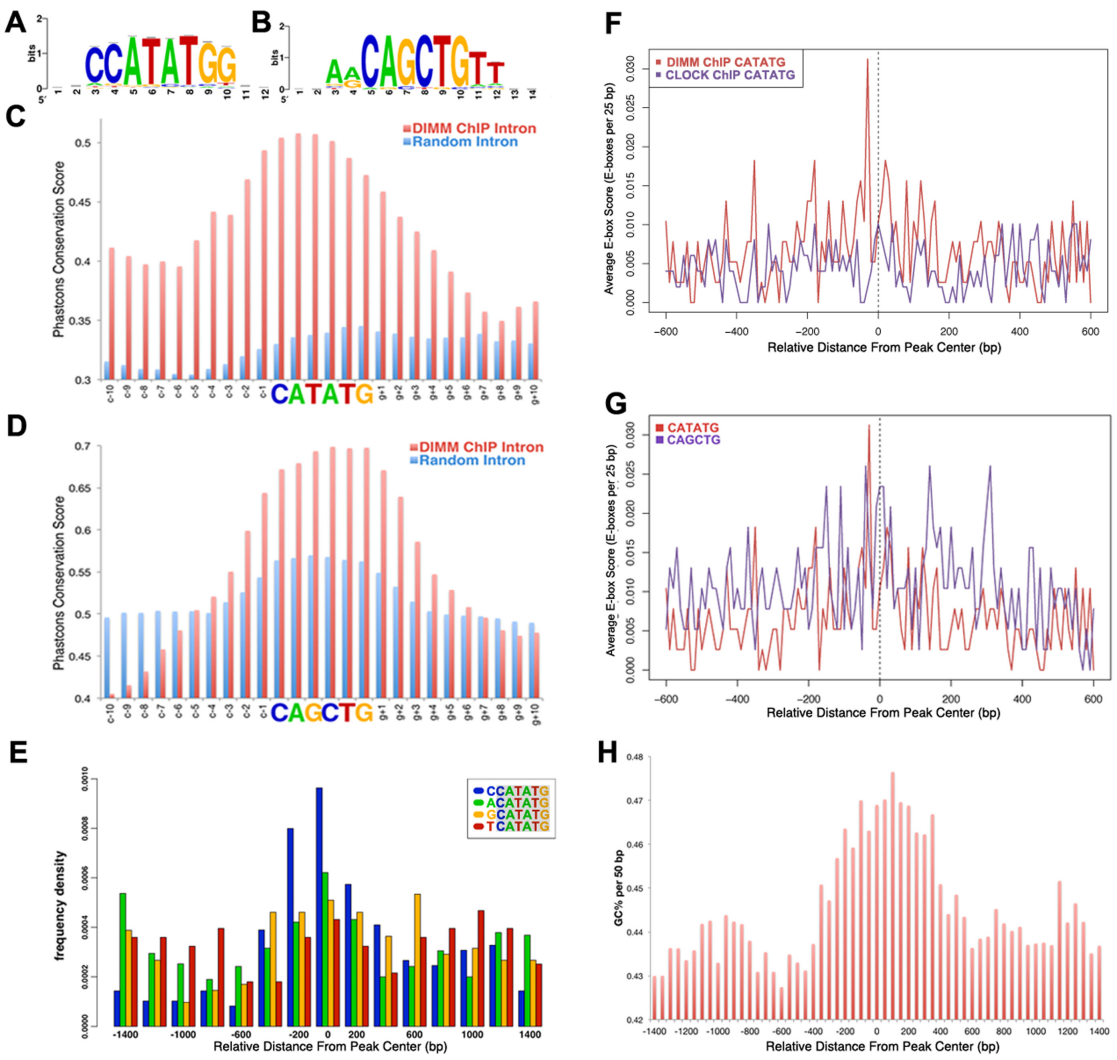
DIMM binds to conserved E-boxes located close to the centers of DIMM ChIP-chip binding peaks. (**A, B**) DIMM preferentially binds to two palindromic E-boxes, CATATG and CAGCTG. Both E-boxes match precisely known DIMM E-boxes in the *Phm* 1st intron enhancer (21) ; the CATATG E-box matches the SELEX-predicted DIMM-binding E-box (63). (**C**) Strand-specific conservation of the CATATG E-box in intronic DIMM binding peaks compared to ∼800 randomly selected intronic CATATG E-boxes. (**D**) Strand-specific conservation of the CAGCTG E-box in intronic DIMM binding peaks compared to ∼800 randomly selected intronic CAGCTG E-boxes. (**E**) The CATATG E-box shows an enrichment for the CCATATG variant, particularly around the centers of binding peaks. (**F**) The CATATG E-box is enriched in the center of DIMM-bound peaks (red) and lacks enrichment in CLOCK-bound peaks (blue). CLOCK data from (26). (**G**) Multiple CATATG and CAGCTG E-boxes are seen dispersed throughout peak length, but they appear concentrated near the center of DIMM-bound peaks. (**H**) DIMM-occupied genomic regions are GC-rich, particularly near the centers.

**Figure 5.**
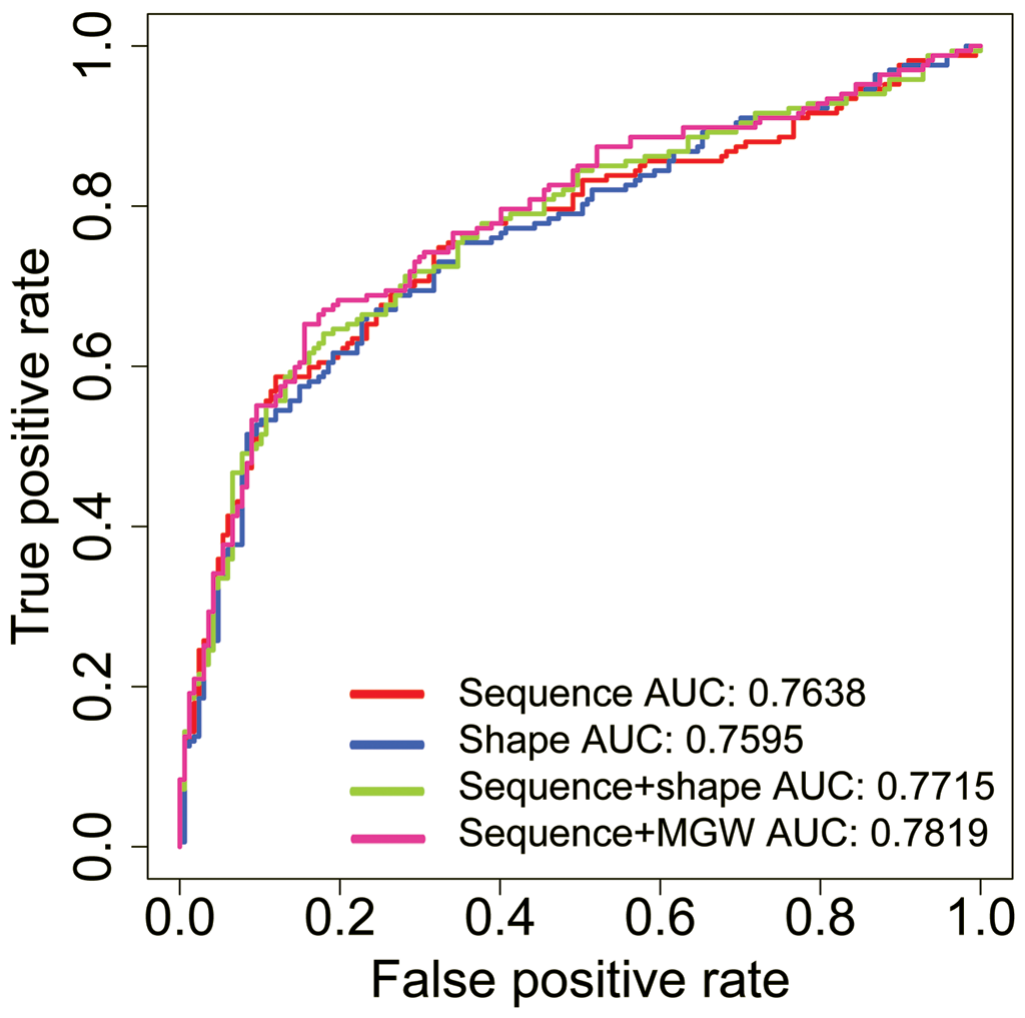
Receiver operating characteristic (ROC) curve of different models for classification of DIMM bound versus unbound putative sites based on DNA sequence and shape features derived from the binding site's flanking regions. Area under the curve (AUC) values were calculated from true positive rates plotted against false positive rates for classification of bound versus unbound sequences containing the CATATG E-box. Different models, including a nucleotide sequence model (red), a DNA shape model (combining minor groove width, roll, propeller twist and helix twist; blue), a shape-augmented sequence model (using both sequence and the four DNA shape features; green) and a sequence-based model augmented by only minor groove width (MGW; magenta), were used to distinguish between bound and unbound DIMM target sites. The AUC values for the CATATG E-box are given in the legend, and the respective values for the CAGCTG E-box were 0.5980, 0.5921, 0.5941 and 0.5999.

**Figure 6.**
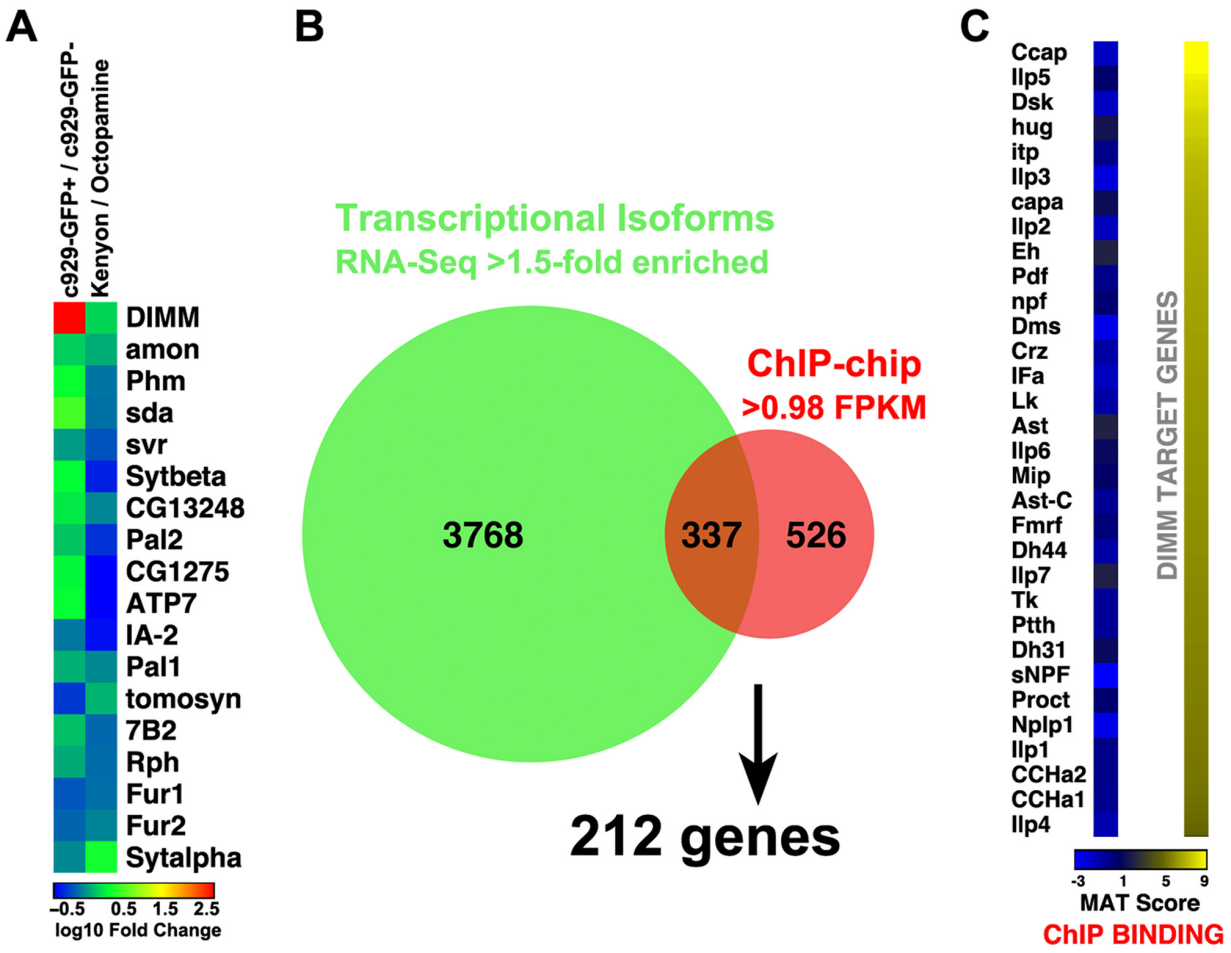
DIMM-binding sites correlate with expression of structural and functional elements of the regulated secretory pathway. (**A**) DIMM binds to a core set of genes encoding biosynthetic, neuropeptide processing enzymes that are highly expressed by *c929*-DIMM^+^ cells. Gene expression from this study compared to RNA-Seq data from (55) obtained from purified nuclei of octopaminergic and Kenyon neurons adult brain neurons. (**B**) Intersection of ChIP-chip identified DIMM target genes and the *c929*-DIMM^+^ cell transcriptome. (**C**) DIMM does not bind to the defined neuropeptide-encoding genes of the *Drosophila* genome.

### DNA sequence and shape analysis

DIMM binding sites were derived from the ChIP-chip peaks and aligned based on their conserved E-box motifs, CATATG or CAGCTG. DNA sequence features were derived from the flanks of these core motifs. DNA shape features (minor groove width, roll, propeller twist and helix twist) were predicted using DNAshape, a high-throughput method for inferring DNA shape parameters from sequence (38). Models combining DNA sequence and shape features were trained using L2-regularized multiple linear regression (MLR) with the assumption that ChIP-chip peaks indicate bound DIMM sites (response variable assigned a value of 1), whereas the random first-intron E-boxes represent unbound sites (response variable assigned a value of 0). The performance of sequence-based, shape-based and sequence+shape classification models was evaluated using area under the curve (AUC) values derived from the receiver operating characteristic (ROC) curves based on 10-fold cross validation.

### *In vitro* luciferase DIMM transactivation assays

We performed luciferase assays as described by Park *et al*. (21), selecting DIMM-bound genomic fragments based on their MAT scores, and in some cases based on their intronic location. Assays were performed with at least *n* = 3 biological replicates.

### Preparation of cells for fluorescence-activated cell sorting

We harvested cells from brains of homozygous *w^1118^*, UAS-*dcr2*; *c929*-GAL4, UAS-*cD8::EGFP*; flies for fluorescence-activated cell sorting (FACS) and subsequent RNA-Seq. This strain is essentially wild-type with respect to DIMM physiology, as evidenced by normal neuropeptide staining (data not shown). Young flies (5–12-days old) grown at 25°C on standard *Drosophila* cornmeal media were anesthetized with carbon dioxide and collected on ice. We dissected brains of 140 (first experiment) and 180 flies (second experiment) within a 2–3-h time period in cold saline, as in Nagoshi *et al*. (39) with some modifications. We used sterile, ice-cold Modified Dissecting Saline (MDS) for dissections (9.9-mM HEPES-KOH buffer, 137-mM NaCl, 5.4-mM KCl, 0.17-mM NaH_2_PO4, 0.22-mM KH_2_PO4, 33-mM glucose, 43.8-mM sucrose, pH 7.4; (40)). Isolated brains were immediately transferred into sterile, chilled modified SM^active^ medium: they were pooled in a 2-ml Eppendorf DNA LoBind nuclease-free tube, then washed with 1 ml of chilled MDS (SM^active^ medium containing 5-mM Bis-Tris; (41)).

Fly brains were then centrifuged at 1000 x g for 30 s in a microcentrifuge. Frozen L-cysteine-activated papain (50 units/ml in dissecting saline; Worthington) was activated at 37°C for 20 min immediately prior to the end of dissection. We then added 450 μl of activated papain to the brains, resuspended them and then incubated with papain for 20 min at room temperature. Papain digestion was quenched by adding 1.5 ml of Schneider's medium supplemented with 1% heat-inactivated Fetal Calf Serum. The sample was centrifuged at 500 x g for 30 s at room temperature. Brains were washed two more times and were finally resuspended in 600 μl of Schneider's medium supplemented with 1% Fetal Calf Serum (FCS).

Papain-digested brains were then triturated 30 times with a flame-rounded P1000 filter tip (medium tip opening), 30 times with a flame-rounded P1000 filter tip (small opening), 30 times with a flame-rounded P200 filter tip (large opening), 40 times with a flame-rounded P200 filter tip (medium tip opening) and 20 times with a flame-rounded P200 filter tip (small opening). Pipette volumes used for trituration were 500 and 150 μl for the P1000 and P200 pipettes, respectively. Trituration efficiency was moderate, such that very small pieces of brain tissue were still visible under a dissection microscope at the end of the procedure.

We strained the brain homogenate through a sterile 70-micron cell strainer (Fisher Sci) mounted on a 50-ml Falcon, then centrifuged briefly at 400 rpm at 4°C. Flow through containing filtered dissociated cells was then strained through a 35-micron sterile cell strainer (BD Falcon) mounted on a 50-ml Falcon, then centrifuged briefly at 200 rpm. 450 μl of dissociated single cells were collected in a 6-ml BD Falcon tube and transported on ice to the Washington University Siteman Flow Cytometry Core for FACS sorting.

### FACS sorting

Cell sorting was performed on a DAKO-Cytomation MoFlo High Speed Sorter at the Siteman Flow Cytometry Core, Washington University in St. Louis. The green fluorescent protein (GFP+) gate was set based on established criteria and experience of the Core's personnel with the sorter. Sorting GFP+ and GFP− cells was completed within 1 h of the start of sorting. Roughly, 1–3% of all cells in the samples were GFP+, which is consistent with the number of *c929*+ cells in the adult fly brain (42). In order to establish the viability of cells after dissociation, a pilot sorting experiment was performed in which cells were sorted for GFP, as well as their ability to exclude the vitality dye 7-Amino-Actinomycin D. This nucleic acid dye allows detection of dying cells by entering such cells due to fragmentation of their membranes (43). The pilot experiment showed that the majority of gated GFP+ and GFP− cells excluded 7-AAD, and were therefore alive at the time of sorting. The fact that sorted DIMM+ cells were viable shortly before RNA capture increased confidence that any subsequent expression profiling would faithfully reproduce the transcriptome of live DIMM+ cells. Because double sorting against GFP and 7-AAD doubled the amount of time needed for sorting, sorting experiments for RNA collection were conducted without 7-AAD. The combined number of cells sorted from the two experiments was 3.0 × 10^5^ GFP+ cells and 1.35 × 10^6^ GFP− cells.

### RNA isolation, processing and illumina HiSeq 2000 sequencing

At the end of sorting, cells were transferred directly into Qiagen's RLT buffer supplemented with beta-mercaptoethanol (Qiagen RNA MinElute kit). Most investigators harvesting nano scale RNA samples for microarray gene expression profiling use the Arcturus PicoPure kit (13,39). This kit contains a poly(dI:dC)-based proprietary nucleic acid carrier embedded in the column. This carrier does not interfere with microarray applications, but could interfere with deep sequencing of RNAs isolated this way. Therefore, we decided to use the Qiagen kit for small samples, which lacks carriers. After transferring cells into RLT buffer, they were vortexed for 6 s, and lysed by passing the suspension five times through a 21.5 gage needle mounted on a 3-ml syringe. RNA was isolated as recommended by Qiagen's MinElute protocol, with the exception of using 65°C-heated water for enhanced RNA recovery. Next, DNA in the sample was digested with the DNase I Turbo DNA-free kit (Ambion). After DNA removal, RNAs isolated from GFP+ and GFP− cells from each of the two experiments were pooled.

Samples with pooled GFP+ and GFP− RNAs were then submitted to the Genome Technology Access Center (Washington University Genetics Department). RNA quality was then checked by Qubit (Invitrogen), NanoDrop (GE Health) and Bioanalyzer (Agilent) quality control assays. Neither sample showed signs of degradation or DNA contamination. Thirty-five nanograms of RNA from GFP+ cells left and 35 ng of RNA from GFP− cells were processed for sequencing on Illumina's HiSeq 2000 platform.

Because Illumina's deep sequencing protocols require 10 μg of RNA, the samples were amplified with the NuGen Ovation RNA-Seq system, a single primer-based RNA amplification product that uses isothermal amplification (44,45). After amplification, the samples were prepared for deep sequencing according to standard Illumina procedures, which included a poly-A selection step and multiplexing. Sequencing was performed in a single lane of an Illumina HiSeq 2000 machine. Base calls were made by Illumina's software (Eland), followed by demultiplexing.

Cufflinks and Tophat algorithm were used to align raw sequence reads to the reference genome and to map splice junctions against *Drosophila* dm3 r5.50 genome release (46,47). Alignments were indexed and sorted for visualization in the Integrated Genomics Viewer (48). Indexed RNA-Seq reads were visualized as coverage tracks. In order to be able to compare samples directly, coverage tracks were always graphed with identical y-axis coordinates.

### ChIP-chip and RNA-Seq data integration

There were a total of 1809 ChIP-chip peak-associated transcripts. 366 transcripts were not expressed in either sequenced sample. We applied a threshold to the data according to absolute gene expression by searching for genes expressed at low levels, that are nevertheless known to be enriched in *c929*-GFP+ cells. The *Pick1* gene (49) displayed a transcriptional isoform expressed at 0.98 FPKMs in the *c929*-GFP+ sample, and 0.26 FPKMs in the *c929*-GFP− sample. It was the lowest expression level we could identify among genes previously identified by independent criteria to be ‘*c929*-cell-enriched’. Therefore, we employed a transcript expression cutoff of 0.98 FPKMs in the *c929*-GFP+ sample throughout this study. In order to establish whether or not the statistical overlap was due solely to chance, we calculated a cumulative hypergeometric probability as follows: population size = 30 459 total transcripts; number of successes in population = 4105 transcripts simultaneously expressed at 0.98 FPKMs or higher in the *c929*-GFP+ sample and enriched at 1.5-fold or higher in the *c929*-GFP+ sample compared to the *c929*-GFP− sample; sample size = 1809 ChIP-chip associated transcripts; number of successes in the sample = 337 ChIP-chip transcripts simultaneously expressed at the level of Pick1 or higher in the *c929*-GFP+ sample and enriched at 1.5-fold or higher in the *c929*-GFP+ sample compared to the *c929*-GFP− sample. Instead of a simple hypergeometric probability, the more stringent cumulative probability of obtaining 337 or more hits due to chance alone was calculated: *P*(*X* ≥ 337) was equal to 9.435933 × 10^−11^. Three hundred and thirty seven ChIP-chip associated transcripts enriched at 1.5-fold or higher with expression level greater or matching that of Pick1 in the *c929*-GFP+ sample yielded a total of 212 genes.

## RESULTS

### The transcriptome of DIMM^+^ NE cells

We targeted *c929*-GAL4^+^ (*c929*^+^) neurons for purification by directing their expression of GFP (genotype – *w^−^*, UAS*-dcr2; c929-*GAL4, UAS*-EGFP*). Figure 1A illustrates GFP expression in the brain of that genotype. Approximately 300 neurons (of a total of ∼50 000 neurons) are GFP^+^ (10). Flies were dissected quickly *en masse*, and brains gently dissociated by mild protease treatment (following the methods of Nagoshi *et al*. (39), with minor modifications—see the Materials and Methods section). Vital dye staining produced no evidence for dying cells among dissociated cells (data not shown); cells were then FACS-sorted to purify DIMM-expressing, GFP^+^ cells away from all GFP^−^ (non-DIMM) cells (Figure 1B). Inspection of purified cell populations at the light level indicated that most/all of the *c929*-GFP^+^ sample had green fluorescence, whereas the *c929*-GFP^−^ sample lacked such (Figure 1C). We predicted that, if this purification method were efficient in capturing intact DIMM*^+^* cells, at least some *c929*-GFP^+^ cells should display LDCVs in their cytoplasm. After FACS isolation, we therefore processed pools of GFP^+^ and GFP^−^ cells for transmission EM and studied >100 cells in each sample (Figure 1D and E). In spite of the extended dissection and dissociation procedure, a substantial fraction (∼10%) of individual *c929*-GFP^+^ cells contained unambiguous LDCVs in their cytoplasm, indicative of a normal NE differentiation (Figure 1D). We found no incidence of LDCV inclusion in the *c929*-GFP^−^ sample (Figure 1E).

We next defined the transcriptional profiles of the two cell samples by deep sequencing with minimal amplification (Supplementary Table S1). Those results confirmed earlier suggestions of accurate sorting and high purity between the two samples: the *c929*-GFP^+^ sample displayed high levels of *dimm* transcription and the *c929*-GFP^−^ sample displayed little to none (Figure 2A). Figure 2A also shows RNA-seq tracks from *Phm* and several other well-defined ‘peptidergic’ loci. *Phm* is the amidating enzyme necessary for neuropeptide biosynthesis for >90% of all *Drosophila* neuropeptides (50), and a ‘bona fide’ direct DIMM target (21). *Phm* is expressed in neurons throughout the CNS, at low, medium and high levels—its highest levels are found in DIMM^+^ NE cells (51). We obtained RNA-Seq data at the *Phm* locus indicating a >4-fold enrichment of *Phm* in DIMM^+^ cells versus that in randomly sorted cells (Figure 2A). Therefore, as predicted, *Phm* expression was enriched in the *c929*-GFP^+^ sample, though it was not exclusive to that cell type.

Different *c929*^+^ cells express diverse neuropeptides, all at high levels (11). RNA-seq results were concordant with those and similar histologic findings from earlier studies, as the *c929^+^* cell transcriptome was highly enriched for different neuropeptide RNAs (Figure 2B). For example, most or all of the NE cells that express *Pigment dispersing factor* (*Pdf*), *eclosion hormone* (*Eh*) and *IFamide* are DIMM^+^: the *c929*-GFP^+^ cells likewise demonstrated a high degree of enrichment of *Pdf, Eh* and *IFa* by RNA-Seq (Figure 2A and B). In contrast, the two identified prothoracicotropic hormone (PTTH) neurons are DIMM^−^, (neuropeptide) proctolin neurons are largely DIMM^−^ and most *tachykinin*-expressing (Tk^+^) neurons are local interneurons and not DIMM^+^ NE cells (52). Likewise, *c929*^+^ neurons were poorly enriched for these transcripts by RNA-Seq (Figure 2B bottom). RNA-Seq specificity was further evident when examining the *Drosophila* insulin-like peptides Ilp2, Ilp3 and Ilp5: they are expressed at high levels in the DIMM^+^ cells of the *Pars intercerebralis* (52), a brain region sometimes analogized with the mammalian hypothalamus (53). Other *Drosophila* insulin genes, such as *Ilp1* and *Ilp4* are not expressed in the CNS at all (reviewed by Nässel *et al*. (54)). Figure 2A shows the *Ilp1-4* locus, with clear enrichment of *Ilp2* and *3*, whereas *Ilp1* and *Ilp4* are not enriched by RNA-Seq. Thus, by a variety of measures, the purified *c929*-GFP^+^ cell population displayed expected enrichment for many different transcripts predicted from previous diverse studies.

We also wanted to compare *c929*-GFP^+^ cells against previously characterized *Drosophila* neuronal cell subpopulations. Figure 2C shows that *c929*-GFP^+^ cells express lower levels of the GABAergic marker *Gad1*, the serotonergic marker *Ddc*, the cholinergic marker *VAchT* and the glutamatergic marker *VGlut* compared to randomly sorted *c929*-GFP^−^ cells. These findings demonstrate that *c929*^+^ cells are transcriptionally distinct from other neuronal subpopulations known to engage predominantly in conventional (i.e. non-peptidergic) neurotransmission.

We also compared the RNA-Seq results of the *c929*^+^ population with those from other purified *Drosophila* adult brain cells, including Kenyon cells, Octopaminergic cells and a generic neuronal population (*elav*^+^) (Figure 6A and Supplementary Figure S1; (55)). Notably, many of the genes that critically support neuropeptide biosynthesis (*amon* (PC2), *svr* (CPD/E) and *Phm*) are all enriched in *c929^+^* neurons, though not exclusively. For example, mRNA levels of key NE proteins like the PC2-associated 7B2, the cytochrome b-561 CG1275 and the copper transporter ATP-7 also display relative high levels in purified octopaminergic cells (Supplementary Figure S1).

### Genome-wide identification of direct DIMM targets by *in vivo* ChIP-chip

To pursue the specific bases of DIMM actions, we next turned to identify and quantify precise sites of DIMM occupancy *in vivo* throughout the genome. For this purpose, we employed a tagged ChIP-chip strategy ((26); see the Materials and Methods section). We directed conditional (i.e. adult-specific) expression of a UAS-*dimm::myc* fusion transgene in adult *c929*^+^ neurons, but only after flies had completed normal larval, pupal and adult development stages (Figure 3A). We compared that genotype to flies of identical genetic constitution, but lacking the *dimm::myc* transgene (Figure 3A). We used a MYC antibody directed against the human MYC hexapeptide tag to detect loci occupied by DIMM::MYC. For each sample, anti-MYC ChIPs were carried out in parallel on both genotypes. We normalized ChIP DNA against input DNA and hybridized four samples per biological replicate, in parallel, to *Drosophila* Affymetrix Tiling 2.0 arrays.

After normalization and averaging, we identified 384 genomic loci that bound DIMM at statistically significant levels (*P*-value ≤1E-04; see the Materials and Methods section) in two independent DIMM ChIP experiments. Descriptions of the loci are provided in Supplementary Table S2. To evaluate the success of the tagged ChIP approach, we examined DIMM occupancy at its known binding site in the first intron of *Phm* (Figure 3E; (11)): indeed, this site displayed statistically significant DIMM binding. To begin the process of identifying additional putative targets, genome-wide, we next assigned annotated genes to each of the 384 DIMM-occupied peaks (see the Materials and Methods section for details). Thus, we could map 384 peaks to 539 genes, albeit with some redundancy, reflecting those cases when adjacent genes overlapped a single peak, and allowing for some peaks that were not situated within 3 kb of an annotated open reading frame. We used the modENCODE classification of chromatin states (identified in the neuronal *BG3* cell line; modENCODE consortium (56)) to determine that the majority of DIMM binding sites fell within areas of active chromatin (Supplementary Figure S4).

DIMM binding was found at genes that represent (i) known DIMM targets such as *Phm, CG1275* and *mael* (Figure 3E): these three were first identified in an embryonic DIMM over-expression screen—Park *et al*. (21)); (ii) targets with clear association with DIMM actions, though not previously defined as DIMM targets, i.e. neuropeptide biosynthetic enzymes, such as PC2 (*amon*—(57)) and carboxypeptidase D/E (*svr*—(58)); as well as (iii) many others genes whose actions can be inferred or remain unknown. We further characterize and provide an overview of DIMM targets below.

Regarding the positions of DIMM binding sites at their target genes, ChIP-chip signals were strikingly enriched near transcription start sites and depleted near transcription termination sites (Figure 3B and C). An analysis that standardized the entire genome to a meta-gene structure showed that these ChIP-chip signals peaked shortly following transcription start sites (Figure 3D). In particular, DIMM bound preferentially to first introns and not to other gene introns (Figure 3F).

### DIMM binds to conserved CATATG and CAGCTG E-boxes with high specificity

After determining that ChIP-chip data corroborated binding to the previously identified DIMM enhancer in *Phm*, we next queried bound sequences for binding sites in an unbiased manner. A motif-calling algorithm identified the CCATATGG motif as the statistically most enriched motif (Figure 4A), with the AACAGCTGTT motif scoring the second highest (Figure 4B). Of the 384 peaks, 271 contained at least one ‘so-called’ ‘TA' or ‘GC' E-box (where TA or GC specifies the two central nucleotides of the canonical CANNTG E-box); 162 of the 271 contained more than one such E-box (Supplementary Table S3). Evolutionary sequence conservation frequently correlates with function: we therefore investigated the degree of conservation of DIMM-bound E-boxes. Compared to E-box sequences chosen from introns of other, ‘random’ euchromatic genes, DIMM-bound CATATG and CAGCTG E-boxes were more conserved (Figure 4C and D). The CATATG E-box was present at half the frequency of the CAGCTG E-box in the fly genome, and appeared to be conserved less, overall. Interestingly, DIMM-bound CATATG E-boxes were quite conserved, and their overall number nearly matched that of CAGCTG E-boxes in DIMM-bound peaks. Furthermore, when the CATATG E-box is examined, DIMM binding peaks showed a higher frequency of CCATATG in their centers compared to other nucleotides (ACATATG, GCATATG and CCATATG; Figure 4E), consistent with our previous findings (11). Individual CATATG and CAGCTG E-boxes were represented multiple times along the length of DIMM-binding regions (Figure 4F and G). We compared DIMM binding to that of CLOCK, a bHLH TF that binds predominantly to the CA**CG**TG E-box (Figure 4F—‘mapped 500’ data from Abbruzzi *et al*. (26)): >90% of DIMM binding sites did not overlap. This general feature of DIMM genomic targets likely corresponds to the finding of multiple functional E-boxes present in the DIMM-sensitive enhancer of *Phm* Intron 1 (11). Finally, we note that DIMM-binding sites are enriched in GC-rich sequences, especially near their centers (Figure 4G). Based on the computational analysis of the putative DIMM-binding E-box, we conclude that DIMM prefers to recognize and bind CATATG and CAGCTG in a highly conserved manner.

### DNA sequence and shape analysis reveals that DIMM binding site flanks contribute to its binding specificity

Increasing evidence suggests that the genomic context of putative binding sites affects *in vivo* TF-DNA binding (59). We therefore studied the effects of regions flanking the core binding sites on the DNA binding preference of DIMM aiming to explain why only a small subset of putative binding sites is actually bound *in vivo*. Specifically, we constructed classification models, based on L2-regularized MLR, that distinguish between bound and unbound DIMM binding sites. We assigned the random first-intron E-boxes (as described above) as unbound sites and E-boxes within ChIP-chip peak regions as bound sites. Whereas the E-box cores of these two groups of target sites were identical in their sequence composition, predictive models that used features derived from flanks of the E-box cores performed better in distinguishing bound from unbound binding sites than a random classification. The model performance was evaluated based on the ROC curve and the calculated AUC. An AUC value of 0.5 indicates a random classifier. For the two types of E-boxes with either the CATATG or CAGCTG core, we compared different models with the goal to identify features that distinguish bound from unbound putative DIMM target sites. These features included DNA sequence and shape features (minor groove width, roll, propeller twist and helix twist), as it was previously shown for bHLH TFs that DNA shape exerts the effect of the genomic flanks on binding specificity (60). The AUC values for the four models based on nucleotide sequence, four shape features, sequence augmented with four shape features and sequence augmented with minor groove width suggested a better classification for the CATACG E-box compared to the CAGCTG E-box (Figure 5). Whereas none of the features could be identified as predominant, taken together, our results show that intrinsic DNA sequence and shape signals encoded in the genomic contexts, such as the flanks, of DIMM binding sites partially explain its *in vivo* DNA binding specificity.

### DIMM can transactivate many of its potential targets

Based on previous evidence, DIMM likely functions as a transcriptional activator (11,21). To support that supposition, we used the *BG3 Drosophila* neuronal cell line and determined if DIMM could transactivate a large number of ChIP-chip-identified regulatory fragments from candidate gene targets. We tested 16 of the 384 DIMM-bound regions in luciferase transactivation assays (Supplementary Figure S2). DIMM transactivated 12 of the 16 candidate regulatory fragments to significant levels. Interestingly, many of the tested enhancers produced inductions that were many fold higher than the strongest inductions recorded previously for target genes *Phm, mael* and *CG6522* (21).

### A high-confidence list of DIMM targets

Having performed two independent assessments of gene expression in *c929*+ cells (ChIP-chip and RNA-Seq), we wished to assess the overlap in gene lists so as to define a high-confidence list of DIMM targets. Here we describe the basis for creating the overlap. As shown in Table 1, ChIP-chip produced 1809 transcripts of interest: these were derived from 539 annotated genes that were adjacent to 384 DIMM-bound genomic loci. From RNA-Seq, we identified 4105 transcripts: these were selected based on two properties. First they represented transcripts enriched >1.5-fold in *c929*+ over *c929*− cell transcriptomes. Secondly, their level of expression exceeded 0.983129 FPKM (Fragments per kilobase of transcript per million mapped reads), which is the level of expression in *c929*+ cells of the gene *Pick1*. Pick1 levels were chosen as a threshold because they displayed the lowest level of expression for any gene known to be enriched in *c929*/DIMM^+^ neurons (49). Comparison of the two groups of transcripts reveals an overlap of 337 isoforms, which translates finally into 212 distinct DIMM target genes (Figure 6B and Supplementary Tables S4 and S5). It is noteworthy that no neuropeptide-encoding genes are on the list due to the lack of DIMM binding (Figure 6C). To provide a context to interpret the targets identified, we categorize them into sets defined by GO functions (Supplementary Table S6). The top three categories include synapse assembly, peptide metabolic process and transport vesicle, which are all terms generally consistent with the concerted actions of DIMM to promote LDCV accumulation.

**Table 1.**
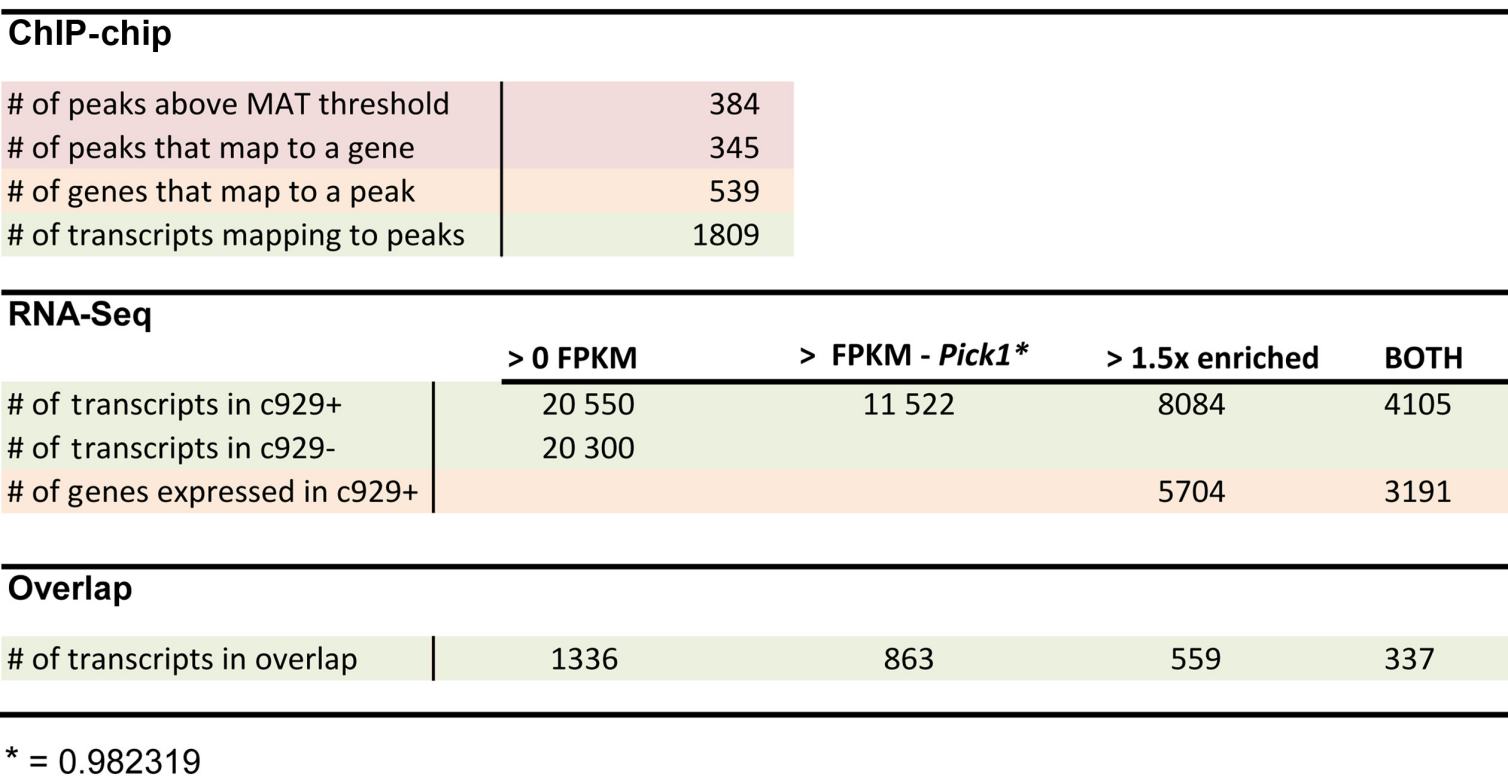
Overview of experimental results

## DISCUSSION

DIMM can orchestrate an active and efficient Regulated Secretory Pathway in NE neurons of *Drosophila*. To deconvolute these processes, we have obtained a broad set of high-confidence DIMM targets. These results identify individual target gene candidates, some of which may explain how DIMM carries out its gene regulation roles. Here we evaluate the quality of the target list and preview ways in which this information can be used to better understand the program of DIMM regulation and the cellular differentiation of Neuroendocrine Neurons.

### Evaluating the quality of the data set

Defining the genome-wide molecular targets for a single TF is subject to both false-negative and false-positive entries. In our studies, false negatives could arise due to the particular developmental stage studied or due to the possibility that DIMM enhancers act at long distance. First we emphasize that the RNA-Seq results argue strongly against a prevalence of false negatives: *dimm* expression was decidedly strong in the *c929*^+^ population, and essentially non-existent in the *c929*^−^ population. Nevertheless, false negatives are an issue because several, previously defined, ‘bona fide’ DIMM targets (like CG13248 and CG17293—(21)) did not appear in the final list of 212 gene targets. The effect of developmental stage is likely significant because the current work was performed on adult tissues, whereas previous efforts were centered on pre-adult stages (11,21). To explain that difference, we propose that some targets may require early, phasic DIMM transactivation, while others may require more sustained activation. Secondly, the effects of long distance enhancers may also contribute to lessening the percentage of true DIMM targets identified here, as we selected candidate genes whose proximal endpoint was less than 3 kb from significant DIMM binding peaks. Inasmuch as DIMM binds to numerous enhancers located within first introns, we are confident that at least a large number of targets were correctly identified, thus minimizing the number of false negatives.

Regarding possible false positives, we consider three sources as most likely: (i) contamination of the two cell populations during sorting, (ii) mis-calling of adjacent genes due to their overlap around a single DIMM binding site or (iii) the use of DIMM over-expression in our experimental design. First, regarding the degree of cell sorting purity, we incorporated ultrastructural analysis of dissociated, FACS-sorted neurons to provide electron microscopic evidence that at least some NE neurons within the population were structurally intact. The percentage of cells displaying LDCV was low, but given the challenging treatment by which cells were purified, and the resulting transcriptional profiling, we submit cell health was not compromised. Second, as we described in Figure 2, predictions of which neuropeptide transcripts should be enriched, and which should not, based on a high degree of cell sorting purity, were strongly supported. Thus we submit that cell mis-sorting provided at most a minor degree of contamination. However, the possibility that we mis-called target genes because they were adjacent to DIMM binding sites, and were also coincidentally enriched in *c929*+ cells, is more difficult to exclude. Future experiments must take this possibility into consideration as individual targets are evaluated. Finally, the risk that identified DIMM binding sites may lack fidelity, due to use of a tagged DIMM over-expression strategy, is a potential concern. We note that Yao *et al*. (61) explicitly compared endogenous and over-expressed MyoD binding sites: they concluded that over-expression of an epitope-tagged MyoD did not alter the profile of normal binding sites. Together these considerations provide confidence that the resulting data set warrants further in-depth interrogation to define the genome-wide program of DIMM's direct molecular actions.

### The DIMM binding site

Previous work demonstrated that DIMM transactivated the amidating enzyme *Phm* by binding to specific E-boxes in its first intron (11). That enhancer harbors several conserved CATATG and CAGCTG E-boxes, three of which (a single–TA- and two –GC-E-boxes) have been shown to be necessary and sufficient *in vitro* and *in vivo* for DIMM transcriptional control (11). Similarly, the DIMM mammalian ortholog (called Mist1) binds to CATATG E-boxes located in the first intron of six of its targets (62). Interestingly, the experimentally identified CCATATGG motif corresponded to the DIMM binding motif discovered by a high throughput bacterial one-hybrid assay (FlyFactorSurvey; (63)): http://pgfe.umassmed.edu/ffs/TFdetails.php?FlybaseID=FBgn0023091). Therefore, the ChIP-chip-identified DIMM-binding E-boxes, derived from analysis of 384 genomic fragments bound by DIMM *in vivo*, matched previously identified E-boxes that DIMM binds to in order to transactivate its targets. This finding increases confidence in the significance of other sites that share these E-box signatures.

### Mechanisms used by DIMM to recognize *in vivo* binding sites

Besides established factors that contribute to TF binding such as chromatin accessibility, methylation state, cofactors and cooperative binding (64), DNA shape was recently shown to exert the influence of genomic sequences that flank TF binding sites on *in vivo* binding (59). Consistent with that hypothesis, our results indicate that DNA shape in flanking regions contributes as much as DNA sequence to DIMM binding. The precise shape features that contribute to the DIMM binding site remain unclear because classification models using nucleotide sequence or DNA shape features performed about equally well in distinguishing bound and unbound target sites. Reasons for the inability to identify any predominant feature could be the limited sample size of a few hundred sequences. Whereas adding shape to sequence slightly improved the performance of the classification model (Figure 5), the additional features could potentially lead to overfitting, which counters a performance increase due to additional information contained in DNA shape features. This hypothesis is supported by the fact that a model that only adds one shape feature, in this case minor groove width (Figure 5), outperformed the sequence+shape model, which uses four different shape features. While more work is needed to identify these precise features, our results indicated that DNA sequence or shape features derived from the E-box flanking sequences play a role in controlling DIMM binding.

### The DIMM gene program

To begin discussing the DIMM program of gene activation we first note that a recently published account of a gene expression screen yielded tens of genes that displayed enriched expression in at least subsets of DIMM/PHM-expressing *Drosophila* neurons (65). Interestingly, five of these genes are also entries on our list of high-confidence DIMM targets: *ERp60, Pfrx, PI3-dependent kinase, hipk* and the RNA-binding protein *alan shepard*. Clearly the DIMM gene program does not feature a simple linear logic whereby a single critical activator is brought to bear. Instead, the present results indicate that DIMM instigates a coordinated program of cellular activities, which we propose are divisible into functional categories.

#### LDCV components

Gene targets that represent LDCV membrane constituents, or which help traffic and accumulate LDCV, are particularly interesting because of DIMM's ability to promote accumulation of LDCV when it is ectopically expressed (17). We submit therefore that the most significant set of DIMM targets is the group of genes encoding LDCV components. First we note five suspected LDCV proteins on our high-confidence target list: ATP7, Cyt-_b_561 (CG1275), CSP and betaTUB56D. ATP7 is a dedicated copper transporter, enriched in NE neurons (66) and critically important for the amidating enzyme PHM, which is a DIMM target and which requires copper as a cofactor (22). CSP is Cysteine String protein, which has been implicated in regulation of the exocytotic event in the membrane fusion of secretory granules (known for its role in regulated exocytosis) (67). We already showed that CSP is upregulated *in vivo* by ectopic DIMM (17) and so its candidacy as a direct DIMM target is validated here. The Jaguar (JAR) protein is an isoform of myosin VI (68), a myosin class that has been implicated in secretory granule exocytosis (69). Along with betaTUB56D, JAR may represent a critical component to permit tethering and accumulation of LDCV in the endings of NE neurons (cf. (70)).

**Figure 7.**
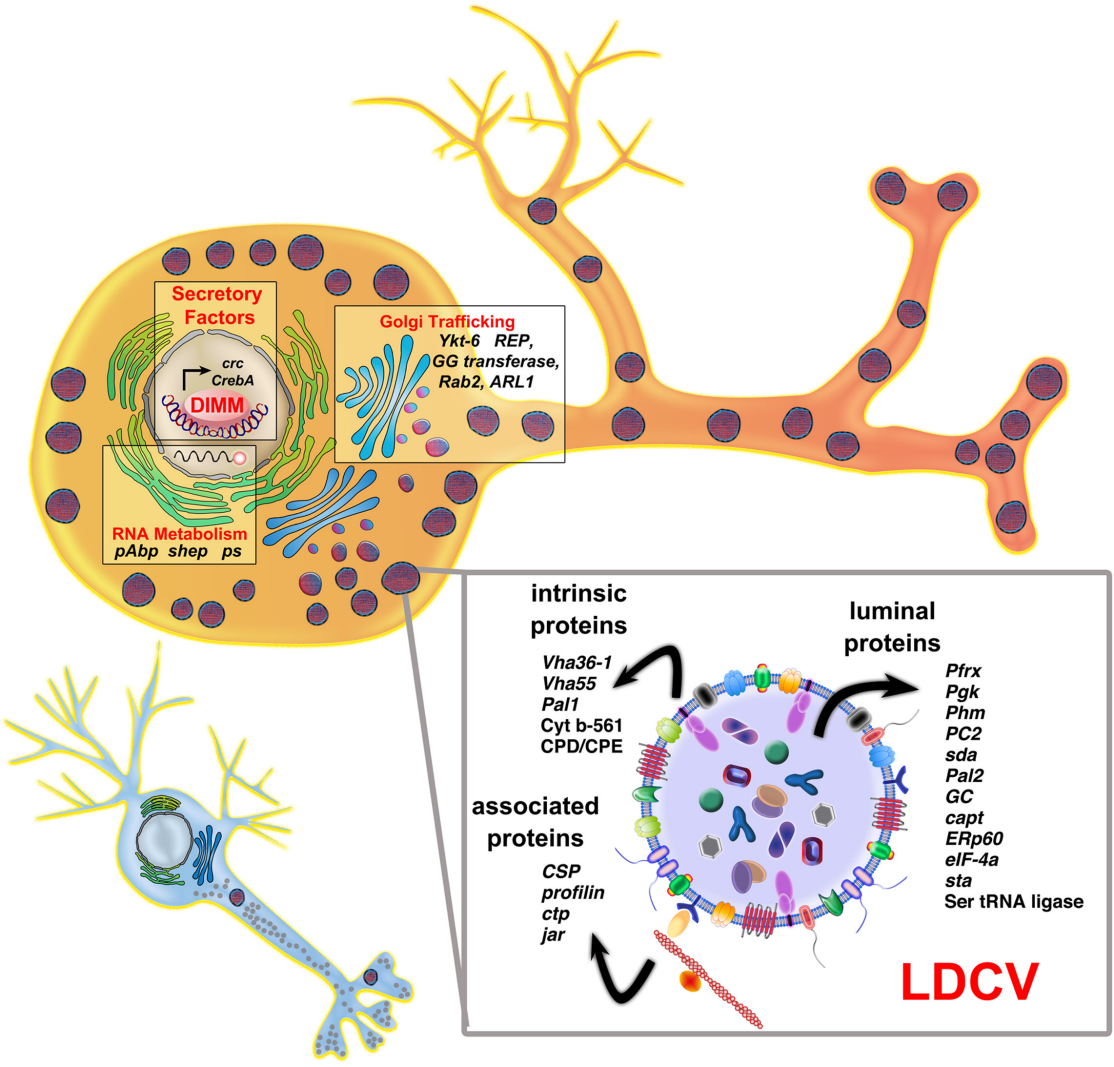
Schematic overview of the DIMM transcriptional output responsible for orchestration of the RSP in NE cells. Illustration of a small conventional neuron (bottom) next to a larger DIMM^+^ NE cell. Various DIMM targets are selected to feature the proximate and distal levels of the secretory pathway that DIMM supports—including nuclear factors that govern specific gene expression, RNA-binding proteins that govern specific translation, Golgi-related factors that regulate the secretory process and LDCV components (in the expanded box).

Extensive proteomics analysis has identified hundreds of proteins resident on human LDCV (35). Gauthier *et al*. (34) identified ∼160 proteins on corticotroph dense core granules, 13 of which remarkably are also found on our list of 212 high value DIMM targets—*Phm, Pal1, Pal2*, amon, seryl *aminoacyl-tRNA synthase 1, EIfalpha48D* (elongation Factor Tu), *Pfrx, Pgk, chic (*Profilin*), eIF-4a, RpS16, RpS2, capt* (adenylate cyclase associated protein1). Two additional DIMM targets are highly related (though not the most highly related *Drosophila* genes) to the corticotroph LDCV proteins prolyl-4-hydrolase *(ERp60)* and a dynein light chain *(ctp*, though it is not in the ‘roadblock’ family of dynein light chains). Thus more than 10% of the DIMM targets here identified are likely components of LDCV as schematized in Figure 7C. These findings argue strongly that the primary mechanism of DIMM action is to promote organization of a stoichiometrically correct and functional secretory granule.

#### Transcription

There are at least 14 TFs included, and several of these also reveal a bias for pro-secretory functions. Notably, CREB-A, a bZIP TF that is especially important as a master regulator of secretory functions in a variety of cell types is a prominent DIMM target. In salivary gland, Andrews *et al*. have shown that CREB-A is a critical coordinating factor in the normal dedication of secretory capacity (7,71), and is a member of the CREB3 family, which appears to have a general role in regulating secretory capacity (72). We interpret the inclusion of CREB-A in the DIMM target list to indicate the co-opting of this secretory program regulator by DIMM to confer its program of high capacity secretory function onto NE cells. DIMM upregulates several other bZIP TFs including ATF-4 (*cryptocephal*), and *vrille* and a few bHLH proteins on the list including *HLH4C, sima* and the orange-domain containing *cwo* (clockwork orange). Several of these have previously been implicated in control of ER capacity in high-level secretory cell types (9,73,74). Finally, we note the transcription factor *HR4* helps shape responsiveness to ecdysteroid pulses (75), which could be a useful feature for NE neurons as they must coordinate their activities closely with the endocrine system that controls developmental timing in insects (e.g. Inka cell physiology).

#### Neuropeptide biosynthesis

Based on many previous results concerning DIMM regulation of *Phm*, it was expected that DIMM should directly target several additional neuropeptide biosynthetic enzymes. The present results strongly confirm this general hypothesis. These enzymes are LDCV proteins (both luminal and membrane components) that help process neuropeptide precursors transiting through the *trans*-Golgi to specific secretory granules (22). DIMM targets many of the genes that encode major enzymes in this pathway, including *Phm* (50), *Pal1* and *Pal2* (76), *amon* (dPC2; (57)) and *silver* (CPD; (58)). Some of these *Drosophila* genes encode multiple isoforms, only some of which operate in the context of neuropeptide biosynthesis. For example, *silver* produces several different RNA isoforms from different promoters that serve both neural and non-neural functions and which have different sub-cellular localizations (77). DIMM transactivates *svr* via the first intron of a specific short isoform whose transcript is enriched in *c929*+ neurons. Another putative neuropeptide biosynthetic enzyme targeted by DIMM is the GC—gamma carboxylase (78)). To date, no *Drosophila* neuropeptides have been identified with a gamma-carboxylate modification.

#### Secretory pathway

The DIMM target list contains numerous factors involved in the Regulated Secretory Pathway and more specifically, in LDCV biogenesis: these include ARF6-GEF *schizo, atl, Arf79F* (the *Drosophila* Arf1/2/6 ortholog), *RhoGEF3* and *RanGAP*. Of special note in this context is the target *Rab2*: it represents a critical point of regulation in the transport of COPI vesicles between the ER and the Golgi. Genetic analyses have shown that Rab2, along with a network of effectors, GAPs and interacting proteins, is required for proper sorting of soluble and trans-membrane LDCV components (79,80,81,82,83). A pair of DIMM targets, REP (Rab escort protein) and l(1)G0144, indicate the importance of Rab prenylation for DIMM functions (84). Thus DIMM, normally orchestrates vesicular trafficking (17), probably in part on the basis of its ability to coordinate expression of the Rab 2 protein and as well two critical components shown to regulate Rab activity. Finally, *Ykt-6* is an intriguing DIMM target: we first identified it in an embryonic screen for DIMM upregulated genes (21). Mammalian Ykt-6 is a prenylated ER-Golgi SNARE, enriched in neurons and partially overlapping with lysosomes and dense-core secretory granules (85,86).

#### Cell metabolism

DIMM neuronal cell bodies are large compared to their non-NE neuronal cohorts and they synthesize large amounts of secretory proteins, by definition. In this regard, Luo *et al*. (87) and Gu *et al*. (88) have recently shown that Insulin Receptor signaling controls DIMM-expressing neuron size; Luo *et al*. (87) find that it does so in a *dimm*-dependent manner and furthermore, that DIMM controls InR levels *in vivo*. Our results suggest that DIMM regulation of *InR* is direct and that it includes coordinate regulation of at least one potential substrate, *rhea*.

A separate class of DIMM targets highlights the role of RNA metabolism in supporting a dedicated pro-secretory phenotype. In particular, polyA BP, pasilla (*ps*) and alan shepherd (*shep*) all are RNA binding proteins with potential broad-scale regulation of the translational capacity of NE cells. Control of specific RNA binding proteins could be a mechanism by which *dimm* regulates neuropeptide mRNA stability. The *ps* gene has previously been implicated in pro-secretory functions in salivary glands, wherein the loss of function phenotype includes a defect of apical secretion (89).

#### A common gene targeted by DIMM and Mist1

Finally, we observe that a single gene is held in common on current lists of gene targets between DIMM and its closest mammalian ortholog, Mist1 (62). Genetic analyses of the two factors suggest they have similar roles supporting secretory activity in serous exocrine cells (Mist1) and in NE cells (DIMM) (14). Tian *et al*. (62) identified 10 Mist1 target genes, and of these, one of the 10, FNDC3A, is also found in the set of high-confidence DIMM targets, defined in the present work (CG42389). This gene encodes a membrane spanning protein with multiple fibronectin domains and is related to signaling proteins like sidekick, DSCAM, and the tyrosine phosphatase DLAR.

### Direct versus indirect DIMM regulation of the NE phenotype

DIMM exhibits the remarkable property of conferring synthesis and accumulation of functional LDCV onto neurons *in vivo* that do not normally display LDCV (17). How it achieves this complex cellular performance at a molecular level motivates our efforts. To that end, we compiled a list of 212 targets using genome-wide methods and the analysis, as discussed above, revealed many genes long-associated with NE function and LDCV biology. However, there are notably several critical ‘NE-related’ genes that are not included in the list of DIMM targets. These are genes whose expression patterns are essentially equal to or overlap with that of DIMM (e.g. *PICK1*—(49,90); *nemy*—(91); *synaptotagmins α* and *β*—(92)). It is possible that some of these are in fact direct DIMM targets and thus represent false negatives in our study. However there is experimental evidence to suggest that, at least for some critical NE proteins, DIMM regulation is substantial, but indirect (93).

## SUPPLEMENTARY DATA

Supplementary Data are available at NAR Online.

SUPPLEMENTARY DATA

## References

[1] Burbach J.P., Luckman S.M., Murphy D., Gainer H. (2001). Gene regulation in the magnocellular hypothalamo-neurohypophysial system. Physiol. Rev..

[2] Helfrich-Forster C., Winter C., Hofbauer A., Hall J.C., Stanewsky R. (2001). The circadian clock of fruit flies is blind after elimination of all known photoreceptors. Neuron.

[3] Rao S., Lang C., Levitan E.S., Deitcher D.L. (2001). Visualization of neuropeptide expression, transport, and exocytosis in Drosophila melanogaster. J. Neurobiol..

[4] Beuret N., Stettler H., Renold A., Rutishauser J., Spiess M. (2004). Expression of regulated secretory proteins is sufficient to generate granule-like structures in constitutively secreting cells. J. Biol. Chem..

[5] Lee A.H., Chu G.C., Iwakoshi N.N., Glimcher L.H. (2005). XBP-1 is required for biogenesis of cellular secretory machinery of exocrine glands. EMBO J..

[6] D'Alessandro R., Meldolesi J. (2013). Expression and function of the dense-core vesicle membranes are governed by the transcription repressor REST. FEBS Lett..

[7] Abrams E.W., Andrew D.J. (2005). CrebA regulates secretory activity in the Drosophila salivary gland and epidermis. Development.

[8] Allan D.W., Park D., St Pierre S.E., Taghert P.H., Thor S. (2005). Regulators acting in combinatorial codes also act independently in single differentiating neurons. Neuron.

[9] Gauthier S.A., Hewes R.S. (2006). Transcriptional regulation of neuropeptide and peptide hormone expression by the Drosophila dimmed and cryptocephal genes. J. Exp. Biol..

[10] Hewes R.S., Park D., Gauthier S.A., Schaefer A.M., Taghert P.H. (2003). The bHLH protein Dimmed controls neuroendocrine cell differentiation in Drosophila. Development.

[11] Park D., Shafer O.T., Shepherd S.P., Suh H., Trigg J.S., Taghert P.H. (2008). The Drosophila basic helix-loop-helix protein DIMMED directly activates PHM, a gene encoding a neuropeptide-amidating enzyme. Mol. Cell. Biol..

[12] Johnson C.L., Kowalik A.S., Rajakumar N., Pin C.L. (2004). Mist1 is necessary for the establishment of granule organization in serous exocrine cells of the gastrointestinal tract. Mech. Dev..

[13] Ramsey V.G., Doherty J.M., Chen C.C., Stappenbeck T.S., Konieczny S.F., Mills J.C. (2007). The maturation of mucus-secreting gastric epithelial progenitors into digestive-enzyme secreting zymogenic cells requires Mist1. Development.

[14] Mills J.C., Taghert P.H. (2012). Scaling factors: transcription factors regulating subcellular domains. BioEssays.

[15] Dannies P.S. (1999). Protein hormone storage in secretory granules: mechanisms for concentration and sorting. Endocr. Rev..

[16] Park J.J., Loh Y.P. (2008). How peptide hormone vesicles are transported to the secretion site for exocytosis. Mol. Endocrinol..

[17] Hamanaka Y., Park D., Yin P., Annangudi S.P., Edwards T.N., Sweedler J., Meinertzhagen I.A., Taghert P.H. (2010). Transcriptional orchestration of the regulated secretory pathway in neurons by the bHLH protein DIMM. Curr. Biol..

[18] Lehman J.J., Barger P.M., Kovacs A., Saffitz J.E., Medeiros D.M., Kelly D.P. (2000). Peroxisome proliferator-activated receptor gamma coactivator-1 promotes cardiac mitochondrial biogenesis. J. Clin. Invest..

[19] Russell L.K., Mansfield C.M., Lehman J.J., Kovacs A., Courtois M., Saffitz J.E., Medeiros D.M., Valencik M.L., McDonald J.A., Kelly D.P. (2004). Cardiac-specific induction of the transcriptional coactivator peroxisome proliferator-activated receptor gamma coactivator-1alpha promotes mitochondrial biogenesis and reversible cardiomyopathy in a developmental stage-dependent manner. Circ. Res..

[20] Scarpulla R.C. (2008). Transcriptional paradigms in mammalian mitochondrial biogenesis and function. Physiol. Rev..

[21] Park D., Hadzic T., Yin P., Rusch J., Abruzzi K., Rosbash M., Skeath J.B., Panda S., Sweedler J.V., Taghert P.H. (2011). Molecular organization of Drosophila neuroendocrine cells by dimmed. Curr. Biol..

[22] Eipper B.A., Milgram S.L., Husten E.J., Yun H.Y., Mains R.E. (1993). Peptidylglycine alpha-amidating monooxygenase: a multifunctional protein with catalytic, processing, and routing domains. Protein Sci..

[23] Menet J.S., Abruzzi K.C., Desrochers J., Rodriguez J., Rosbash M. (2010). Dynamic PER repression mechanisms in the Drosophila circadian clock: from on-DNA to off-DNA. Genes Dev..

[24] McGuire S.E., Le P.T., Osborn A.J., Matsumoto K., Davis R.L. (2003). Spatiotemporal rescue of memory dysfunction in Drosophila. Science.

[25] Johnson W.E., Li W., Meyer C.A., Gottardo R., Carroll J.S., Brown M., Liu X.S. (2006). Model-based analysis of tiling-arrays for ChIP-chip. Proc. Natl. Acad. Sci. U.S.A..

[26] Abruzzi K.C., Rodriguez J., Menet J.S., Desrochers J., Zadina A., Luo W., Tkachev S., Rosbash M. (2011). Drosophila CLOCK target gene characterization: implications for circadian tissue-specific gene expression. Genes Dev..

[27] Goecks J., Nekrutenko A., Taylor J. (2010). Galaxy: a comprehensive approach for supporting accessible, reproducible, and transparent computational research in the life sciences. Genome Biol..

[28] Liu T., Ortiz J.A., Taing L., Meyer C.A., Lee B., Zhang Y., Shin H., Wong S.S., Ma J., Lei Y. (2011). Cistrome: an integrative platform for transcriptional regulation studies. Genome Biol..

[29] Shin H., Liu T., Manrai A.K., Liu X.S. (2009). CEAS: cis-regulatory element annotation system. Bioinformatics.

[30] Thomas-Chollier M., Defrance M., Medina-Rivera A., Sand O., Herrmann C., Thieffry D., van Helden J. (2011). RSAT 2011: regulatory sequence analysis tools. Nucleic Acids Res..

[31] Siepel A., Bejerano G., Pedersen J.S., Hinrichs A.S., Hou M., Rosenbloom K., Clawson H., Spieth J., Hillier L.W., Richards S. (2005). Evolutionarily conserved elements in vertebrate, insect, worm, and yeast genomes. Genome Res..

[32] Zambon A.C., Gaj S., Ho I., Hanspers K., Vranizan K., Evelo C.T., Conklin B.R., Pico A.R., Salomonis N. (2012). GO-Elite: a flexible solution for pathway and ontology over-representation. Bioinformatics.

[33] Saito R., Smoot M.E., Ono K., Ruscheinski J., Wang P.L., Lotia S., Pico A.R., Bader G.D., Ideker T. (2012). A travel guide to Cytoscape plugins. Nat. Methods.

[34] Gauthier D.J., Sobota J.A., Ferraro F., Mains R.E., Lazure C. (2008). Flow cytometry-assisted purification and proteomic analysis of the corticotropes dense-core secretory granules. Proteomics.

[35] Bark S.J., Wegrzyn J., Taupenot L., Ziegler M., O'Connor D.T., Ma Q., Smoot M., Ideker T., Hook V. (2012). The protein architecture of human secretory vesicles reveals differential regulation of signaling molecule secretion by protein kinases. PloS one.

[36] Lyne R., Smith R., Rutherford K., Wakeling M., Varley A., Guillier F., Janssens H., Ji W., McLaren P., North P. (2007). FlyMine: an integrated database for Drosophila and Anopheles genomics. Genome Biol..

[37] St Pierre S.E., Ponting L., Stefancsik R., McQuilton P. (2014). FlyBase 102—advanced approaches to interrogating FlyBase. Nucleic Acids Res..

[38] Zhou T., Yang L., Lu Y., Dror I., Dantas Machado A.C., Ghane T., Di Felice R., Rohs R. (2013). DNAshape: a method for the high-throughput prediction of DNA structural features on a genomic scale. Nucleic Acids Res..

[39] Nagoshi E., Sugino K., Kula E., Okazaki E., Tachibana T., Nelson S., Rosbash M. (2010). Dissecting differential gene expression within the circadian neuronal circuit of Drosophila. Nat. Neurosci..

[40] Jiang S.A., Campusano J.M., Su H., O'Dowd D.K. (2005). Drosophila mushroom body Kenyon cells generate spontaneous calcium transients mediated by PLTX-sensitive calcium channels. J. Neurophysiol..

[41] Kuppers-Munther B., Letzkus J.J., Luer K., Technau G., Schmidt H., Prokop A. (2004). A new culturing strategy optimises Drosophila primary cell cultures for structural and functional analyses. Dev. Biol..

[42] Park D., Taghert P.H. (2009). Peptidergic neurosecretory cells in insects: organization and control by the bHLH protein DIMMED. Gen. Comp. Endocrinol..

[43] Schmid I., Hausner M.A., Cole S.W., Uittenbogaart C.H., Giorgi J.V., Jamieson B.D. (2001). Simultaneous flow cytometric measurement of viability and lymphocyte subset proliferation. J. Immunol. Methods.

[44] Dafforn A., Chen P., Deng G., Herrler M., Iglehart D., Koritala S., Lato S., Pillarisetty S., Purohit R., Wang M. (2004). Linear mRNA amplification from as little as 5 ng total RNA for global gene expression analysis. BioTechniques.

[45] Kurn N., Chen P., Heath J.D., Kopf-Sill A., Stephens K.M., Wang S. (2005). Novel isothermal, linear nucleic acid amplification systems for highly multiplexed applications. Clin. Chem..

[46] Roberts A., Pimentel H., Trapnell C., Pachter L. (2011). Identification of novel transcripts in annotated genomes using RNA-Seq. Bioinformatics.

[47] Trapnell C., Pachter L., Salzberg S.L. (2009). TopHat: discovering splice junctions with RNA-Seq. Bioinformatics.

[48] Robinson J.T., Thorvaldsdottir H., Winckler W., Guttman M., Lander E.S., Getz G., Mesirov J.P. (2011). Integrative genomics viewer. Nat. Biotechnol..

[49] Jansen A.M., Nassel D.R., Madsen K.L., Jung A.G., Gether U., Kjaerulff O. (2009). PICK1 expression in the Drosophila central nervous system primarily occurs in the neuroendocrine system. J. Comp. Neurol..

[50] Kolhekar A.S., Mains R.E., Eipper B.A. (1997). Peptidylglycine alpha-amidating monooxygenase: an ascorbate-requiring enzyme. Methods Enzymol..

[51] Taghert P.H., Hewes R.S., Park J.H., O'Brien M.A., Han M., Peck M.E. (2001). Multiple amidated neuropeptides are required for normal circadian locomotor rhythms in Drosophila. J. Neurosci..

[52] Park D., Veenstra J.A., Park J.H., Taghert P.H. (2008). Mapping peptidergic cells in Drosophila: where DIMM fits in. PloS one.

[53] Hartenstein V. (2006). The neuroendocrine system of invertebrates: a developmental and evolutionary perspective. J. Endocrinol..

[54] Nassel D.R., Kubrak O.I., Liu Y., Luo J., Lushchak O.V. (2013). Factors that regulate insulin producing cells and their output in Drosophila. Front. Physiol..

[55] Henry G.L., Davis F.P., Picard S., Eddy S.R. (2012). Cell type-specific genomics of Drosophila neurons. Nucleic Acids Res..

[56] Roy S., Ernst J., Kharchenko P.V., Kheradpour P., Negre N., Eaton M.L., Landolin J.M., Bristow C.A., Ma L., Lin M.F. (2010). Identification of functional elements and regulatory circuits by Drosophila modENCODE. Science.

[57] Siekhaus D.E., Fuller R.S. (1999). A role for amontillado, the Drosophila homolog of the neuropeptide precursor processing protease PC2, in triggering hatching behavior. J. Neurosci..

[58] Sidyelyeva G., Baker N.E., Fricker L.D. (2006). Characterization of the molecular basis of the Drosophila mutations in carboxypeptidase D. Effect on enzyme activity and expression. J. Biol. Chem..

[59] Gordân R., Shen N., Dror I., Zhou T., Horton J., Rohs R., Bulyk M.L. (2013). Genomic regions flanking E-box binding sites influence DNA binding specificity of bHLH transcription factors through DNA shape. Cell Rep..

[60] Yang L., Zhou T., Dror I., Mathelier A., Wasserman W.W., Gordân R., Rohs R. (2014). TFBSshape: a motif database for DNA shape features of transcription factor binding sites. Nucleic Acids Res..

[61] Yao Z., Fong A.P., Cao Y., Ruzzo W.L., Gentleman R.C., Tapscott S.J. (2013). Comparison of endogenous and overexpressed MyoD shows enhanced binding of physiologically bound sites. Skeletal Muscle.

[62] Tian X., Jin R.U., Bredemeyer A.J., Oates E.J., Blazewska K.M., McKenna C.E., Mills J.C. (2010). RAB26 and RAB3D are direct transcriptional targets of MIST1 that regulate exocrine granule maturation. Mol. Cell. Biol..

[63] Zhu L.J., Christensen R.G., Kazemian M., Hull C.J., Enuameh M.S., Basciotta M.D., Brasefield J.A., Zhu C., Asriyan Y., Lapointe D.S. (2011). FlyFactorSurvey: a database of Drosophila transcription factor binding specificities determined using the bacterial one-hybrid system. Nucleic Acids Res..

[64] Slattery M., Zhou T., Yang L., Dantas Machado A.C., Gordân R., Rohs R. (2014). Absence of a simple code: how transcription factors read the genome. Trends Biochem. Sci..

[65] Chen D., Qu C., Hewes R.S. (2014). Neuronal remodeling during metamorphosis is regulated by the alan shepard (shep) gene in Drosophila melanogaster. Genetics.

[66] Sellami A., Wegener C., Veenstra J.A. (2012). Functional significance of the copper transporter ATP7 in peptidergic neurons and endocrine cells in Drosophila melanogaster. FEBS Lett..

[67] Graham M.E., Burgoyne R.D. (2000). Comparison of cysteine string protein (Csp) and mutant alpha-SNAP overexpression reveals a role for csp in late steps of membrane fusion in dense-core granule exocytosis in adrenal chromaffin cells. J. Neurosci..

[68] Kisiel M., Majumdar D., Campbell S., Stewart B.A. (2011). Myosin VI contributes to synaptic transmission and development at the Drosophila neuromuscular junction. BMC Neurosci..

[69] Bond L.M., Peden A.A., Kendrick-Jones J., Sellers J.R., Buss F. (2011). Myosin VI and its binding partner optineurin are involved in secretory vesicle fusion at the plasma membrane. Mol. Biol. Cell.

[70] Bulgari D., Zhou C., Hewes R.S., Deitcher D.L., Levitan E.S. (2014). Vesicle capture, not delivery, scales up neuropeptide storage in neuroendocrine terminals. Proc. Natl. Acad. Sci. U.S.A..

[71] Fox R.M., Hanlon C.D., Andrew D.J. (2010). The CrebA/Creb3-like transcription factors are major and direct regulators of secretory capacity. J. Cell Biol..

[72] Barbosa S., Fasanella G., Carreira S., Llarena M., Fox R., Barreca C., Andrew D., O'Hare P. (2013). An orchestrated program regulating secretory pathway genes and cargos by the transmembrane transcription factor CREB-H. Traffic.

[73] Ameri K., Harris A.L. (2008). Activating transcription factor 4. Int. J. Biochem. Cell Biol..

[74] Hewes R.S., Schaefer A.M., Taghert P.H. (2000). The cryptocephal gene (ATF4) encodes multiple basic-leucine zipper proteins controlling molting and metamorphosis in Drosophila. Genetics.

[75] Ou Q., Magico A., King-Jones K. (2011). Nuclear receptor DHR4 controls the timing of steroid hormone pulses during Drosophila development. PLoS Biol..

[76] Han M., Park D., Vanderzalm P.J., Mains R.E., Eipper B.A., Taghert P.H. (2004). Drosophila uses two distinct neuropeptide amidating enzymes, dPAL1 and dPAL2. J. Neurochem..

[77] Kalinina E., Fontenele-Neto J.D., Fricker L.D. (2006). Drosophila S2 cells produce multiple forms of carboxypeptidase D with different intracellular distributions. J. Cell. Biochem..

[78] Jakubowski J.A., Kelley W.P., Sweedler J.V. (2006). Screening for post-translational modifications in conotoxins using liquid chromatography/mass spectrometry: an important component of conotoxin discovery. Toxicon.

[79] Ailion M., Hannemann M., Dalton S., Pappas A., Watanabe S., Hegermann J., Liu Q., Han H.F., Gu M., Goulding M.Q. (2014). Two Rab2 interactors regulate dense-core vesicle maturation. Neuron.

[80] Hannemann M., Sasidharan N., Hegermann J., Kutscher L.M., Koenig S., Eimer S. (2012). TBC-8, a putative RAB-2 GAP, regulates dense core vesicle maturation in Caenorhabditis elegans. PLoS Genet..

[81] Sumakovic M., Hegermann J., Luo L., Husson S.J., Schwarze K., Olendrowitz C., Schoofs L., Richmond J., Eimer S. (2009). UNC-108/RAB-2 and its effector RIC-19 are involved in dense core vesicle maturation in Caenorhabditis elegans. J. Cell Biol..

[82] Buffa L., Fuchs E., Pietropaolo M., Barr F., Solimena M. (2008). ICA69 is a novel Rab2 effector regulating ER-Golgi trafficking in insulinoma cells. Eur. J. Cell Biol..

[83] Edwards S.L., Charlie N.K., Richmond J.E., Hegermann J., Eimer S., Miller K.G. (2009). Impaired dense core vesicle maturation in Caenorhabditis elegans mutants lacking Rab2. J. Cell Biol..

[84] Charng W.L., Yamamoto S., Jaiswal M., Bayat V., Xiong B., Zhang K., Sandoval H., David G., Gibbs S., Lu H.C. (2014). Drosophila Tempura, a novel protein prenyltransferase alpha subunit, regulates notch signaling via Rab1 and Rab11. PLoS Biol..

[85] Hasegawa H., Zinsser S., Rhee Y., Vik-Mo E.O., Davanger S., Hay J.C. (2003). Mammalian ykt6 is a neuronal SNARE targeted to a specialized compartment by its profilin-like amino terminal domain. Mol. Biol. Cell.

[86] Cooper A.A., Gitler A.D., Cashikar A., Haynes C.M., Hill K.J., Bhullar B., Liu K., Xu K., Strathearn K.E., Liu F. (2006). Alpha-synuclein blocks ER-Golgi traffic and Rab1 rescues neuron loss in Parkinson's models. Science.

[87] Luo J., Liu Y., Nassel D.R. (2013). Insulin/IGF-regulated size scaling of neuroendocrine cells expressing the bHLH transcription factor Dimmed in Drosophila. PLoS Genet..

[88] Gu T., Zhao T., Hewes R.S. (2014). Insulin signaling regulates neurite growth during metamorphic neuronal remodeling. Biol. Open.

[89] Seshaiah P., Miller B., Myat M.M., Andrew D.J. (2001). pasilla, the Drosophila homologue of the human Nova-1 and Nova-2 proteins, is required for normal secretion in the salivary gland. Dev. Biol..

[90] Holst B., Madsen K.L., Jansen A.M., Jin C., Rickhag M., Lund V.K., Jensen M., Bhatia V., Sorensen G., Madsen A.N. (2013). PICK1 deficiency impairs secretory vesicle biogenesis and leads to growth retardation and decreased glucose tolerance. PLoS Biol..

[91] Iliadi K.G., Avivi A., Iliadi N.N., Knight D., Korol A.B., Nevo E., Taylor P., Moran M.F., Kamyshev N.G., Boulianne G.L. (2008). nemy encodes a cytochrome b561 that is required for Drosophila learning and memory. Proc. Natl. Acad. Sci. U.S.A..

[92] Adolfsen B., Saraswati S., Yoshihara M., Littleton J.T. (2004). Synaptotagmins are trafficked to distinct subcellular domains including the postsynaptic compartment. J. Cell Biol..

[93] Park D., Li P., Dani A., Taghert P.H. (2014). Peptidergic cell-specific synaptotagmins in Drosophila: Localization to dense core granules and regulation by the bHLH protein DIMMED. J. Neurosci..

