# Supplementary material for: Genome-wide features of neuroendocrine regulation in Drosophila by the basic helix-loop-helix transcription factor DIMMED
Source: Nucleic Acids Res. 2015 Jan 29;43(4):2199–215. doi: 10.1093/nar/gku1377 (PMC4344488; doi:10.1093/nar/gku1377)
Supplement: SUPPLEMENTARY DATA [file supp_43_4_2199__index.html]

Genome-wide features of neuroendocrine regulation in Drosophila by the basic helix-loop-helix transcription factor DIMMED — SUPPLEMENTARY DATA 

# Genome-wide features of neuroendocrine regulation in *Drosophila* by the basic helix-loop-helix transcription factor DIMMED

## SUPPLEMENTARY DATA

**Files in this Data Supplement:**

- SUPPLEMENTARY DATA
- SUPPLEMENTARY DATA
- SUPPLEMENTARY DATA
- SUPPLEMENTARY DATA
- SUPPLEMENTARY DATA
- SUPPLEMENTARY DATA
- SUPPLEMENTARY DATA
