# Supplementary material for: Genome-wide features of neuroendocrine regulation in Drosophila by the basic helix-loop-helix transcription factor DIMMED
Source: Nucleic Acids Res. 2015 Jan 29;43(4):2199–215. doi: 10.1093/nar/gku1377 (PMC4344488; doi:10.1093/nar/gku1377)

Supplementary Figure and Table Legends

Figure S1. Deep sequencing indicates that a "core" peptidergic gene set is enriched in purified DIMM^{+} neurons. A subset of this gene battery is also enriched in octopaminergic neurons (*Pal2, CG1275, 7B2, ATP7*; data sourced from Henry et al. (1).

Figure S2. **Testing DIMM transactivation in a *Drosophila BG3-c2* neuronal cell line of 16 genomic fragments that displayed significant DIMM *in vivo* ChIP-chip binding.** Luciferase reporter was placed downstream of a mini-*SV* promoter and a ChIP-chip identified enhancer. Fold ratios represent Luciferase levels with DIMM co-transfection divided by those without. Red gene candidates are those producing significant changes: all except the *Vha36-1* fragment responded to DIMM with increases in LUC expression. Histograms represents means and SEMs of at least three independent replicate assays. *$p < 0.05$ student t-test.

Figure S3. *Phm* locus with the normalization steps used in ChIP-chip analysis. MYC antibody ChIP of *c929>DIMM::MYC,tubGAL80^{ts}* fly heads normalized to its input indicated in pink (#1-#2). MYC antibody ChIP of *c929>tubGAL80^{ts}* control fly heads normalized to its input indicated in blue (#3-#4). MYC antibody ChIP of *c929>DIMM::MYC,tubGAL80^{ts}* fly heads normalized directly to MYC antibody ChIP of *c929>tubGAL80^{ts}* control fly heads depicted in orange. Net result summarized by the following equation: [(#1 – #2) – (#3 – #4)] + (#1 – #3) depicted in red. Scales for the pink, blue and orange tracks are identical (range 0-7 MAT score). Scale for the red track (net result) has a range of 0-14 MAT score.

Figure S4. **DIMM ChIP-chip peaks lie mostly in active chromatin.** Percent of peak length mapped against the 9-state chromatin model derived by modENCODE from BG3 cells. Chromatin states 1, 2 and 3 are predominantly found in expressed genes.

Figure S5. The flanking regions of DIMM-bound E-boxes showed differences in DNA sequence and shape features. DNA shape variation due to flanks surrounding intronic CATATG (top) and CAGCTG (bottom) E-boxes selected preferentially by DIMM (white) compared to a set of randomly chosen intronic E-boxes (grey). DNA shape predictions, here shown for minor groove width (MGW), were derived from our DNAshape method (2). Asterisks (*) indicate positions with significant differences ($p < 0.05$, Kolmogorov-Smirnov test) in MGW between the sequences bound by DIMM and randomly chosen E-boxes. The box plots show the range between the 1st and 3rd quartiles, the line within each box indicates the median, and the whiskers define the furthest data points within 1.5 times the interquartile range from the edges of the box. The central six positions represent the E-box core binding site.

Table S1. 30,459 transcripts in purified DIMM+/*c929*+ and DIMM-/*c929*- cells. See attached Excel file.

Table S2. 384 DIMM ChIP-chip peaks statistically significant at p-value $\leq 1 \times 10^{-4}$ with assigned 539 genes.

Table S3. E-boxes found within DIMM-occupied statistically significant genomic regions.

Table S4. 337 DIMM-associated transcripts expressed at or above *Pick1* in the *c929+* sample and enriched above 1.5-fold in the *c929+* sample compared to the *c929-* sample.

Table S5. 212 DIMM-associated genes with at least one transcript expressed at or above *Pick1* in the *c929+* sample and enriched above 1.5-fold in the *c929+* sample compared to the *c929-* sample. Genes arranged in alphabetical order.

Table S6. GO Elite-derived gene ontology (GO) terms for 212 DIMM-associated genes.

Supplementary References

1. Henry, G.L., Davis, F.P., Picard, S. and Eddy, S.R. (2012) Cell type-specific genomics of *Drosophila* neurons. *Nucleic Acids Res.*, **40**, 9691-9704.

2. Zhou, T., Yang, L., Lu, Y., Dror, I., Dantas Machado, A.C., Ghane, T., Di Felice, R. and Rohs, R. (2013) DNAshape: a method for the high-throughput prediction of DNA structural features on a genomic scale. Nucleic Acids Res., 41, W56–W62.

Figure S1. Deep sequencing indicates that a “core” peptidergic set is enriched in purified DIMM+ neurons.

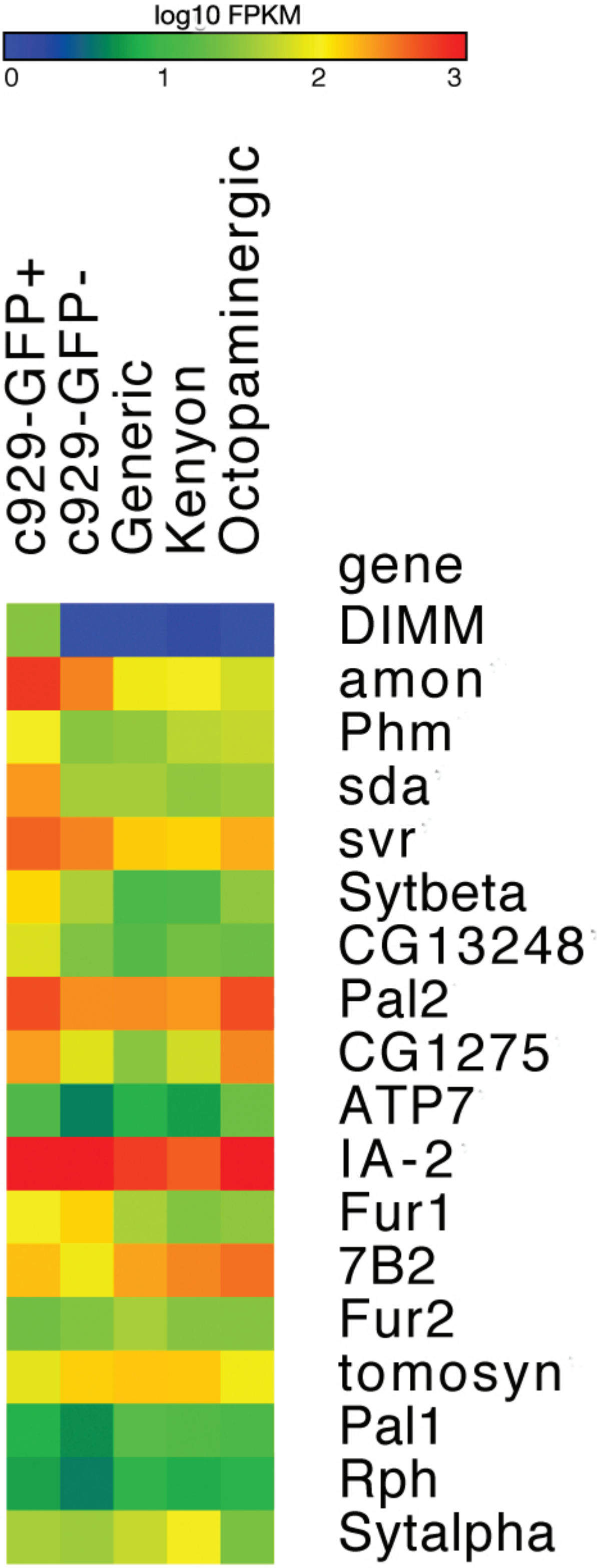

Figure S2. Testing DIMM transactivation in a *Drosophila BG3-c2* neuronal cell line of 16 genomic fragments that displayed significant DIMM *in vivo* ChIP-chip binding.

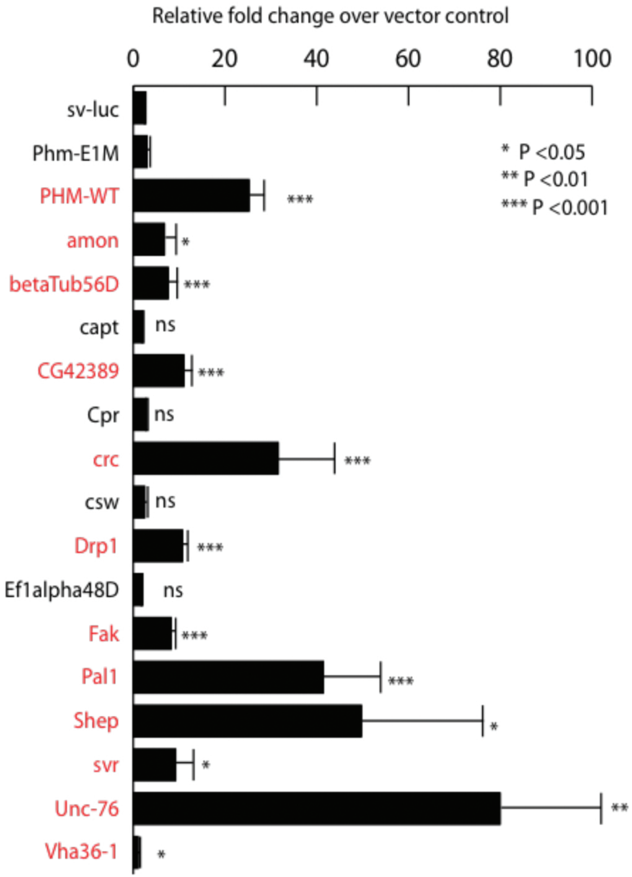

Figure S3. Phm locus with the normalization steps used in ChIP-chip analysis.

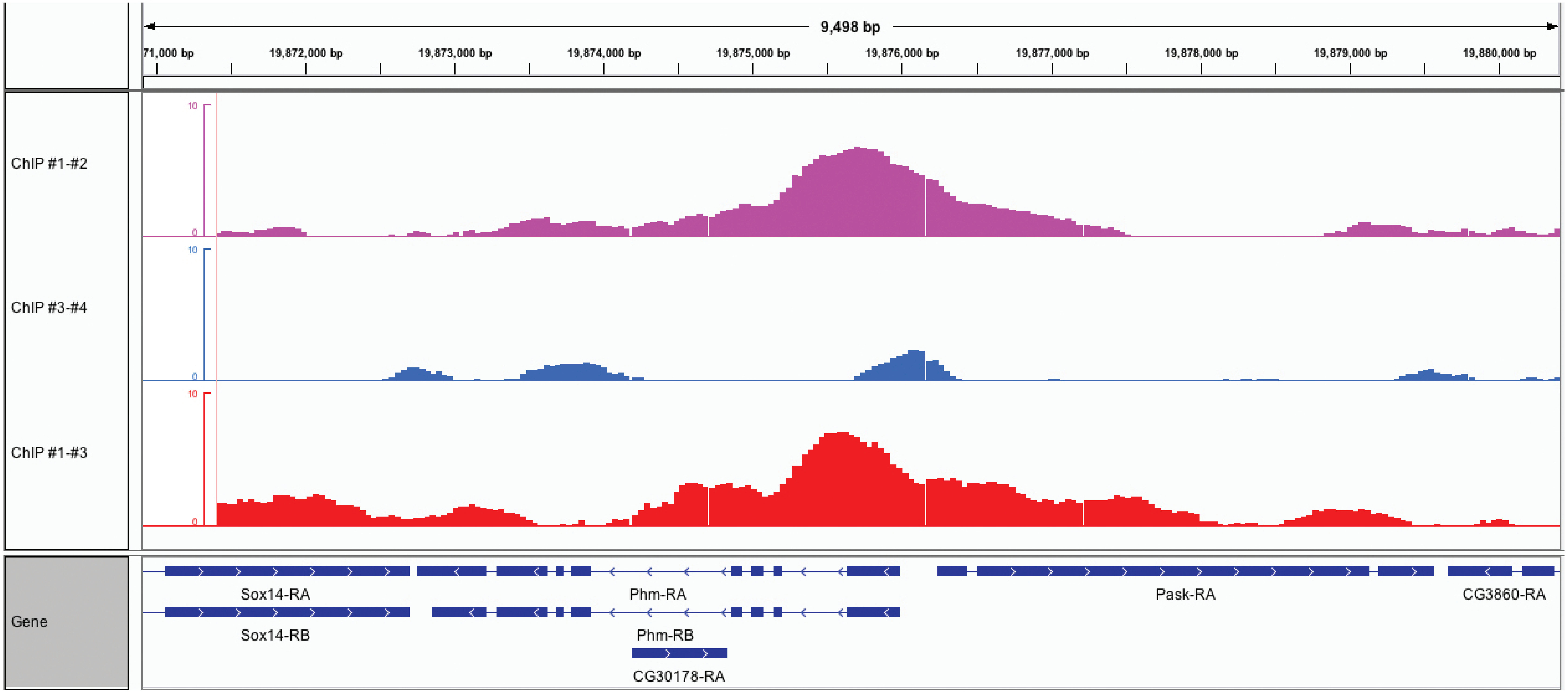

Figure S4. DIMM ChIP-chip peaks lie mostly in active chromatin.

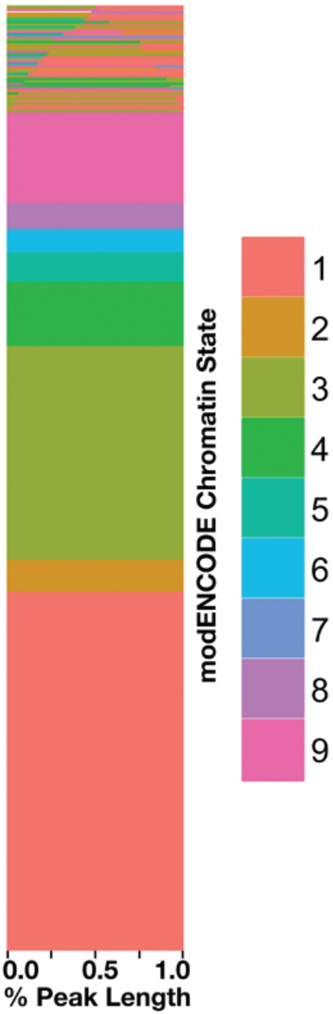

Figure S5. The flanking regions of DIMM-bound E-boxes showed differences in DNA sequence and shape features.

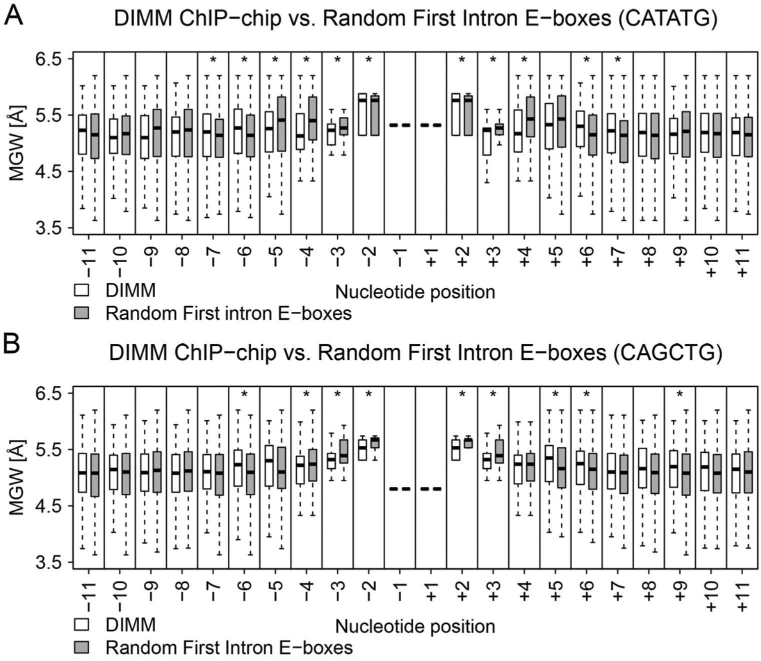

Supplement: SUPPLEMENTARY DATA [file supp_gku1377_Hadzic-et-al-12-14-NAR-Supporting-Information-rr.pdf]
